# Auxin Import and Local Auxin Biosynthesis Are Required for Mitotic Divisions, Cell Expansion and Cell Specification during Female Gametophyte Development in *Arabidopsis thaliana*

**DOI:** 10.1371/journal.pone.0126164

**Published:** 2015-05-13

**Authors:** Aneesh Panoli, Maria Victoria Martin, Monica Alandete-Saez, Marissa Simon, Christina Neff, Ranjan Swarup, Andrés Bellido, Li Yuan, Gabriela C. Pagnussat, Venkatesan Sundaresan

**Affiliations:** 1 Department of Plant Biology, University of California Davis, Davis, California, 95616, United States of America; 2 PIPRA, University of California Davis, Davis, California, 95616, United States of America; 3 Institute of Biological Research IIB-CONICET, Universidad Nacional de Mar del Plata, 7600, Mar del Plata, Argentina; 4 University of Nottingham, Nottingham, United Kingdom; 5 Department of Plant Sciences, University of California Davis, Davis, California, 95616, United States of America; Universidad Miguel Hernández de Elche, SPAIN

## Abstract

The female gametophyte of flowering plants, called the embryo sac, develops from a haploid cell named the functional megaspore, which is specified after meiosis by the diploid sporophyte. In Arabidopsis, the functional megaspore undergoes three syncitial mitotic divisions followed by cellularization to form seven cells of four cell types including two female gametes. The plant hormone auxin is important for sporophytic developmental processes, and auxin levels are known to be regulated by biosynthesis and transport. Here, we investigated the role of auxin biosynthetic genes and auxin influx carriers in embryo sac development. We find that genes from the *YUCCA/TAA* pathway (*YUC1*, *YUC2*, *YUC8*, *TAA1*, *TAR2*) are expressed asymmetrically in the developing ovule and embryo sac from the two-nuclear syncitial stage until cellularization. Mutants for *YUC1* and *YUC2* exhibited defects in cell specification, whereas mutations in *YUC8*, as well as mutations in *TAA1* and *TAR2*, caused defects in nuclear proliferation, vacuole formation and anisotropic growth of the embryo sac. Additionally, expression of the auxin influx carriers *AUX1* and *LAX1* were observed at the micropylar pole of the embryo sac and in the adjacent cells of the ovule, and the *aux1 lax1 lax2* triple mutant shows multiple gametophyte defects. These results indicate that both localized auxin biosynthesis and auxin import, are required for mitotic divisions, cell expansion and patterning during embryo sac development.

## Introduction

The plant life cycle alternates between a diploid (2n) sporophytic and a haploid (n) gametophytic generation. The male gametophyte (pollen) produces the male gametes (two sperm cells), and the female gametophyte (embryo sac) produces the egg cell and central cell, two female gametes that participate in double fertilization to produce a diploid embryo and a triploid endosperm respectively. The development of the female gametophyte (embryo sac) follows a tightly regulated program, which initiates with meiosis and terminates upon fertilization ([1–3]. In Arabidopsis, female meiosis is initiated by the megaspore mother cell (MMC) in the nucellus of the ovule. The MMC undergoes meiosis giving rise to four megaspores, of which the three distal spores will degenerate, while the surviving spore becomes the functional megaspore (FG1, S1 Fig). The haploid functional megaspore undergoes mitosis to generate a 2-nucleate coenocyte (FG2), which is followed by migration of nuclei to opposite poles of the cell and formation of a central vacuole (FG3). A second round of mitosis produces a 4-nucleate embryo sac (FG4) with a large central vacuole and a pair of nuclei at either pole. A characteristic of the FG4 embryo sac is the rapid expansion of its size as well as that of the central vacuole. A final round of mitosis, followed by coordinated nuclear migration, produces an 8-nucleate and highly polarized embryo sac, composed by 3 nuclei occupying the micropylar pole, 3 at the chalazal pole, and 2 lying close to the micropylar end of the central vacuole (FG5). Cellularization results in acquisition of distinct cell fates and the formation of a 7-celled, 8-nucleate embryo sac, composed of 2 synergids, 1 egg cell, 1 central cell with 2 nuclei called polar nuclei, and 3 antipodal cells (S1 Fig, FG6), while the two polar nuclei of the central cell fuse to form the diploid central cell (S1 Fig, FG7) [2,3].

Although relatively inconspicuous, the embryo sac is indispensable for seed formation, and therefore plays a critical role in plant reproduction (reviewed in [4]). It was observed that manipulation of levels of the hormone auxin results in changes in cell fate, with high auxin levels promoting synergid fate or egg cell fates of the antipodal cells, and suppression of auxin signaling promoting egg cell fate in the synergid cells [5,6]. A model was proposed that different concentrations of auxin within the embryo sac might determine cell fates, with the highest auxin concentration-present at the micropylar pole- would specify the synergids, the next lowest specifies the egg cell, while the lowest auxin concentration at the chalazal pole results in antipodal specification. A recent paper though questioned this idea, as mathematical models could not generate a robust auxin gradient, and additionally, the expression of auxin reporters was not found inside the embryo sac [7]. However, and in agreement with a role of auxins during female gametogenesis, it was reported that the auxin efflux carrier PIN1 is required in the maternal sporophytic tissues of the ovule that surround the embryo sac to promote female gametophyte development [8]. Thus, PIN1 is thought to be involved in auxin flux towards the embryo sac as previously suggested [5] and such auxin flux seems essential for gametophyte progression [8]. Furthermore, AUX1, a member of the *AUXIN1/LIKE-AUX1 (AUX/LAX)* family of transporters that mediate auxin influx in *Arabidopsis*, was shown to be expressed inside the female gametophyte and the protein to accumulate in the micropylar pole of the embryo sac [7]. Also, the expression of two auxin biosynthetic genes of the *YUCCA* family was previously observed at the micropylar end of embryo sacs at early stages of development [5]. Thus, both auxin import and local auxin biosynthesis might be involved in gametophyte development.

The pathways leading to auxin biosynthesis in sporophyte development have been studied extensively [9–13] and it is known that auxin can be synthesized locally in several tissues in response to developmental or environmental cues [10,12–14]. Recently, a simple two-step pathway that converts tryptophan to indole-3-acetic acid has been shown to be the main auxin biosynthesis pathway in Arabidopsis [14–16]. Trp is first converted to indole-3-pyruvate (IPA) by the TAA family of amino transferases, and IPA is subsequently converted into IAA by the YUC family of flavinmonooxygenases. Auxin can also be synthesized from indole-3-acetaldoxime (IAOx), which is produced from Trpcatalyzed by CYP79B2 and its close homologue CYP79B3, but it is believed that IAOx may not be a major auxin biosynthetic pathway in plants [17].

Here we have investigated the roles of both auxin import and biosynthesis during female gametophyte development. We found that several genes from the *YUCCA/TAA* pathway are expressed in the developing embryo sac from the two-nuclear syncitial stage until cellularization and that expression of these genes is asymmetrically localized towards the micropylar end of the developing gametophyte. Genetic analysis revealed that mutants for these genes showed female gametophyte defects that range from abnormal cell specification to defects in nuclear proliferation, vacuole formation and cell expansion. Additionally, *AUX1* and *LAX1* expression were detected at the micopylar region of the ovule during female gametophyte development, and the triple mutant for three influx carriers *aux1 lax1 lax2* shows mitotic arrest during female gametogenesis. These results indicate that female gametophyte development requires both localized auxin biosynthesis and auxin import from the sporophytic ovule.

## Results

### *YUCCA* and *TAA/TAR* auxin biosynthetic genes are expressed in the ovule and developing female gametophyte

**Fig 1:**
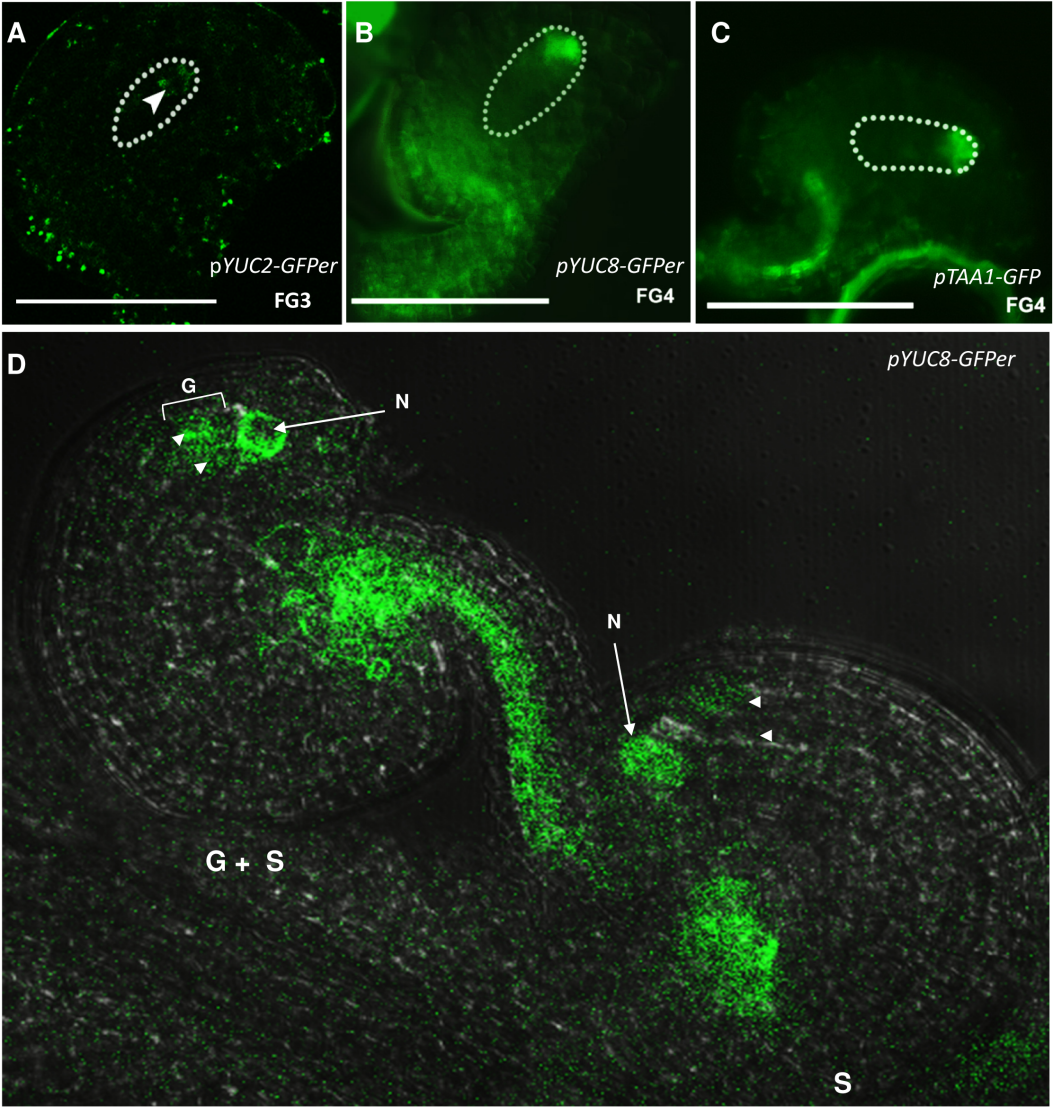
Expression pattern of auxin biosynthetic genes in the developing embryo sac. A, p*YUC2-GFPer* expression at FG3 stage. B, *pYUC8-GFPer* expression in the gametophyte at FG4 stage. C, *pTAA1* expression at FG4 stage of gametophyte development. D, Segregation of GFP signal in a line hemizygous for *pYUC8*::*GFPer*. From the two ovules shown, only one presents GFP detectable inside the embryo sac (marked as G + S). S, indicates sporophytic signal; G, indicates gametophytic signal. N, nucellus. Arrowheads point at embryo sac nuclei.

We previously found that the auxin biosynthetic *YUCCA* genes, *YUC1* and *YUC2*, were expressed sporophytically, and subsequently gametophytically, at the micropylar pole of the developing embryo sac, at FG1-FG2 stages [5]. In this study, we first performed a screen to identify all possible members of the *YUC* gene family that could be involved in embryo sac development. Transgenic plants carrying promoter::GUS fusions as well as promoter::GFPer fusions for the *YUC* genes *YUC1* through *YUC11*, were used to monitor their expression at various stages of embryo sac development. For the 11 *YUC* genes studied, we found only three genes showing consistent visible expression in the developing embryo sac: *YUC1*, *YUC2* and *YUC8* (Fig 1, S2 Fig). As no previous studies reported the expression of *YUC8* in the ovule, its expression pattern was further characterized. *YUC8* expression is the most delayed of the *YUC* genes, with strong expression at the micropylar pole of the gametophyte at the FG3 stage that persists until the FG6 stage (S2A, S2B and S2C Fig). *YUC8* expression is not restricted to the gametophyte, as strong expression can also be detected in the tip of inner and outer integuments as well at FG5 and FG6 stages (S2 Fig). To verify whether *YUC8* expression was indeed gametophytic, the segregation of the GFP signal inside the embryo sac was analyzed in a line hemizygous for *pYUC8*::*GFPer*. In these plants, the ovules at FG5-6 stages segregated for embryo sac signal at a ratio of ~1:1 (45.4% GFP+ vs 54.6% GFP-, N = 196, compared to a homozygous plant showing expression in <90% of the ovules examined, N = 298, Fig 1, S1 Movie). Additionally, as the strongest expression found inside the embryo sac at early stages corresponded to *YUC2*, we decided to further confirm the gametophytic expression by studying the segregation of the GFP signal in a line hemizygous for *pYUC2*::*GFPer*. In these plants, the GFP expressing ovules at FG3 stage segregated at a ratio of 1:1 (44% GFP+ *vs*. 53.4% GFP-, N = 240, compared to a homozygous plant showing expression in ~90% of the ovules examined, N = 400, S2 Movie). A small fraction of the ovules (3.6%) showed expression in the nucellar region, around FG2 stage.

Additionally, the expression of *TAA/TAR* genes was also studied. TAA/TAR were originally thought to act in an alternate auxin pathway, but have subsequently been found to act upstream of the YUC proteins [15,16,18]. Analysis of promoter activity using p*TAA1*::*GFP* and p*TAA1*::*GUS* lines indicated strong expression of *TAA1* at the micropylar pole of the embryo sac from FG3 onwards (Fig 1 and S2G–S2I Fig). The expression starts out as a faint signal at FG3 building up to a strong signal as the embryo sac reaches FG5 stage, persisting beyond this stage to FG6. At FG6 stage, we also detected *TAA1* expression within the endothelial cells of the ovule adjacent to the micropylar region of the embryo sac (S2 Fig). *pTAA1* is also active in the inner integuments and funiculus from FG0 to FG3. Using a p*TAR2*::*GUS* fusion, we found that *TAR2* is also expressed at the micropylar end of the embryo sac, starting slightly later than *TAA1*, at about the FG4 stage (Fig 1). *TAR2* is expressed in the tip of the inner integument as well from the FG5 through FG7 stages. We were not able to detect any *TAR1* expression in the developing ovules, either in the sporophytic tissues or the embryo sac, using a p*TAR1*::*GUS* fusion [10,12–14].

### Loss of *YUC1* and *YUC2* functions affects specification of micropylar cells

**Fig 2:**
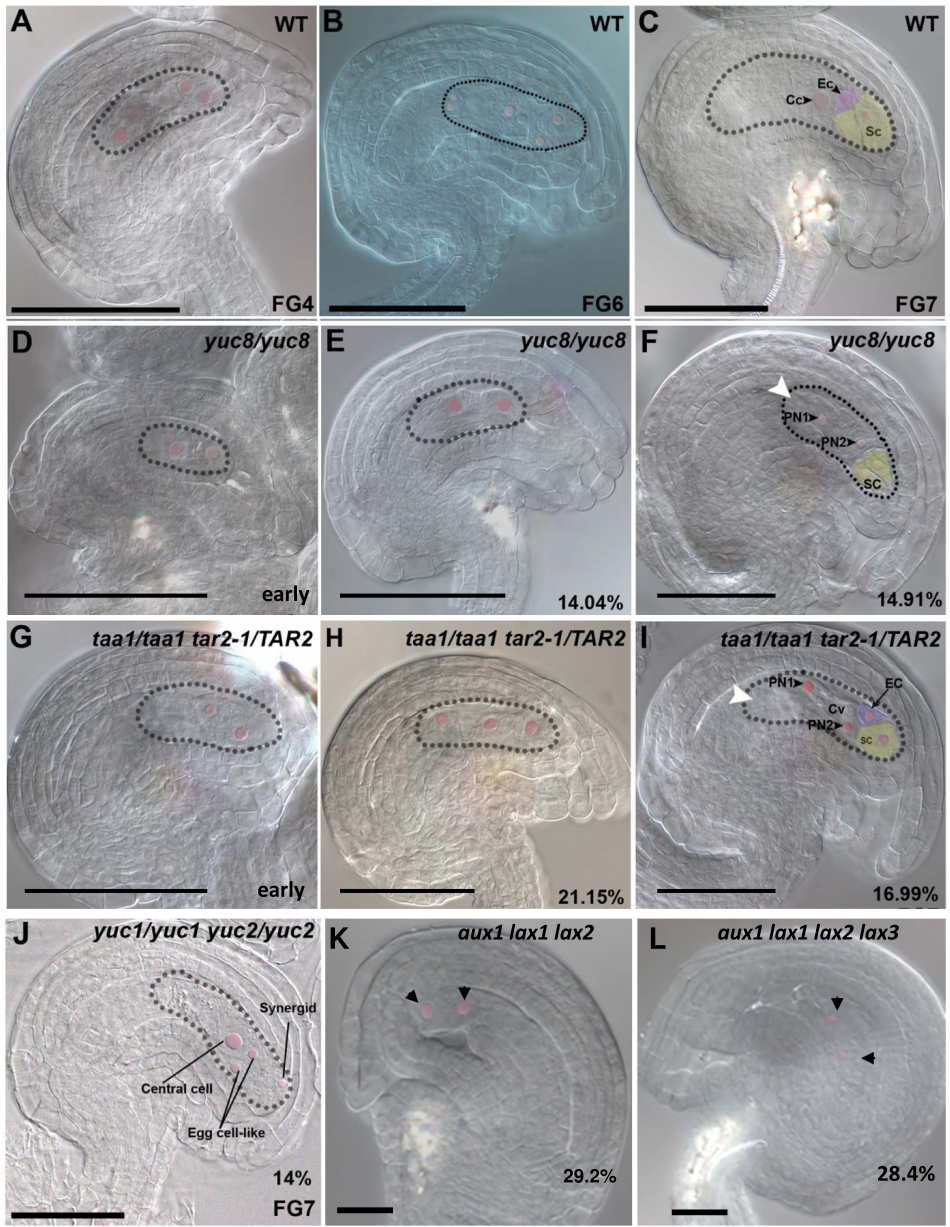
Embryo sac developmental defects in mutants impaired in auxin biosynthesis and import. **A-C,** WT; **D-F,** *yuc8/yuc8*; **G-I,** *taa1/taa1 tar2/TAR2*; **J,** *yuc1/yuc1 yuc2/yuc2*; *aux1 lax1 lax2*; **K**, *aux1 lax1 lax2 lax3*. **A**, WT 4-nucleate embryo sac at FG4 stage. **B,** WT 8-nucleate embryo sac prior to cellularization. **C,** WT mature 4-celled embryo sac *yuc8* mutant embryo sac at FG7 stage showing defects in polar nucleus migration. **D,** *yuc8* mutant gametophyte at FG4 stage containing only 2 nuclei. A comparable WT gametophyte will typically carry 2 nuclei at this stage. **E,** *yuc8* mutant ovule at FG5 stage with a 2-nucleate arrested embryo sac. **F,** Mature *yuc8*mutant embryo sac showing defective polar nucleus migration, no antipodals are visible at this stage. **G,** A mutant ovule, with only 2 nuclei in the coenocytic embryo sac. **H,** Mutant ovule with 3-nucleate gametophyte. Unlike clearly polarized nuclei in the WT, these nuclei are scattered in the cytoplasm. **I,** Mature mutant embryo sac showing defective polar nucleus migration, antipodals are completely degenerated by this stage. **J,** A *yuc1 yuc2* double mutant showing miss-polarized micropylar nuclei, giving rise 2 egg cell-like structures instead of 1. **K**, Ovule from a triple mutant *aux1 lax1 lax2* showing an embryo sac arrested at FG2 stage. The arrows point at the nuclei inside the embryo sac. **L**, Ovule from a quadruple mutant *aux1 lax1 lax2 lax3* showing a collapsing embryo sac containing only two nuclei (arrows). Except for the phenotypes shown in panels D and G, all mutant phenotypes shown are terminal and observed at late stages of ovule development as indicated in Table 1 and in S1 Table. Cv, central vacuole; Ec, egg cell; PN1, polar nucleus 1; PN2, polar nucleus 2; Sc, synergid cell. Scale bar, 50 μM for A-J and 20 μM for K-L.

To uncover the contributions of individual *YUC* genes to female gametophyte development, we examined insertional mutants in *YUC* genes expressed in the embryo sac. The earliest gametophytically expressed *YUC* genes are *YUC1* and *YUC2*. Single mutants in either gene had no detectable defects in embryo sac development. As the overlapping expression patterns of *YUC1* and *YUC2* might result in functional redundancy, we examined *yuc1 yuc2* double mutants. Although sporophytic development appeared normal in the *yuc1 yuc2* mutant plants, marked defects were visible in embryo sac development. In a mature wild-type embryo sac, nuclei of synergid cells usually take up a distal position close to the micropylar end, while the nucleus of the egg cell occupies a somewhat more proximal position (S1 Fig, FG6 and FG7). In *yuc1 yuc2* mutant plants, DIC analysis of emasculated pistils showed 14% of embryo sacs (N = 220) with mis-positioning of the nuclei within the cells at the micropylar end, with two cells containing nuclei at the normal position of the egg cell and only one at the synergid position (Fig 2J). In contrast, wild-type ovules exhibited only 1% of the embryo sacs (N = 550) with this defect. The *yuc1 yuc2* double mutant displayed 94% normal seeds, 1% aborted seeds and 5% undeveloped ovules (N = 447). These numbers are very similar to what is observed in wild-type plants indicating that the *yuc1 yuc2* mutations did not affect the seed set significantly.

**Fig 3:**
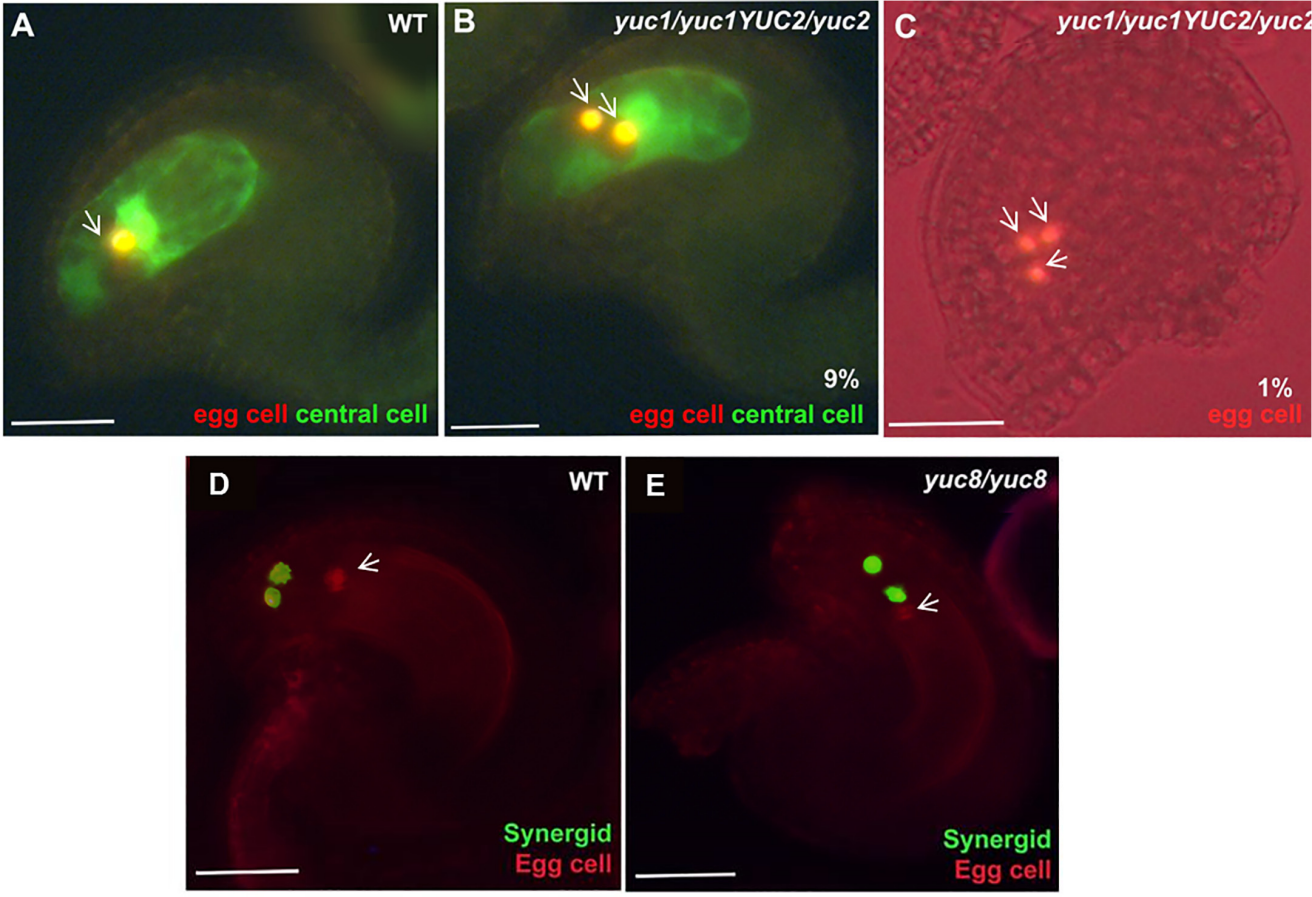
Changes in auxin homoeostasis lead to altered cell specification. **A, D,** WT. **B and C,** *yuc1/yuc1 yuc2/YUC2*. **E,** *yuc8/yuc8*.**A-C,** green signal indicates the expression of central cell specific marker, and red indicates egg cell specific marker. The yellow color observed results from the overlapping of the red colour (egg or egg-like nuclei) on the green background of the central cell in the confocal image. **D-E,** green signal indicates synergid cell-specific marker, and red indicates egg cell-specific marker. **A,** WT ovule showing a single egg cell (Red) and a central cell (green) at FG7 stage. **B,** A mutant ovule at FG7 stage showing 2 egg cells and 1 central cell. **C,** an FG7 mutant ovule showing 3 egg cells. **D,** A WT ovule showing normal polarization of synergid nuclei towards the micropylar end. **E,** merged image of a *yuc8* mutant ovule, showing miss-positioned synergids. The white arrows indicate the egg cell marker expression. Scale bar, 20 μM.

To determine whether cell-specification inside the female gametophyte was affected in the mutant background, we used the specific egg cell marker FGR 1.0- dsRED together with a central cell-specific YFP reporter. In the *yuc1 yuc2* homozygous mutant, embryo sacs with more than one cell expressing the egg cell marker were found (Fig 3B and 3C). The frequency of *yuc1 yuc2* embryo sacs containing multiple cells expressing the egg cell dsRED marker was 10% (N = 227), comprised of 9% showing dsRED expression in two micropylar cells, and 1% showing dsRED expression in all three micropylar cells. The dsRED expressing nuclei were often located at the normal egg cell position as they were seen clustered in between the normal central cell and synergid nuclear positions. Central cell specification, as monitored by expression of the central-cell marker, was unaffected in *yuc1 yuc2* mutant. We never detected any type of cell specification changes using these marker lines in the wild-type gametophytes examined (N = 850), indicating that the synergid to egg cell specification defects arise from the *yuc1 yuc2* mutations.

### Loss of *YUC8* function affects mitotic divisions, cell expansion and nuclear migration during embryo sac development

Our studies using *pYUC8*::*GUS* and *pYUC8*::*GFPer* showed that *YUC8* is the primary *YUC* gene expressed from FG3 to FG6 stages of embryo sac development. Therefore, we examined the *yuc8* mutant for possible gametophytic functions. Homozygous *yuc8* plants were recovered from an F2 population indicating that the mutation can be transmitted through the gametophytes. Sporophytic development appeared normal and ovules displayed proper initiation and growth of integuments. However, cleared ovules showed that *yuc8/YUC8* and *yuc8/yuc8* homozygous mutants are defective in the female gametophyte development (Fig 2D–2F). Mutant gametophytes looked normal from FG1 to FG3 stages of development, consistent with the absence of wild-type *YUC8* expression at these stages. Female gametophyte defects were visible from stage FG4 onwards. Due to significantly delayed gametophyte development in the mutant, we have used ovule stages defined according to Schneitz et al [19] to make comparisons between wild-type and mutant embryo sacs when necessary. The observed phenotypes can be divided into 3 classes, i) defects in mitotic division, ii) defective embryo sac expansion and vacuole development, and iii) defects in nuclear migration. All the mutant gametophytes had a combination of 2 or more defects belonging to the above described phenotypic classes. Gametophytes showing the first class of defects exhibited a delayed gametophyte development as compared to normal ovules. A wild-type ovule at stage *3-IV* usually contains a 4-nucleate FG4 gametophyte (Fig 2A), while all *yuc8* homozygous ovules at stage*3-IV* contained only 2 or 3 nucleate embryo sacs (Fig 2D, N >200). By the *3-V* ovule stage, the *yuc8* mutant displayed a delayed development in 14% of the gametophytes (N = 228), with 2 to 4 large nuclei, compared to wild type with distinct 8-nucleate FG5 embryo sacs (Fig 2E, S1 Table). The size of the nuclei was abnormally larger than those of wild type embryo sacs. In wild-type, the embryo sac usually attains near-maximum size by FG5, accompanied by rapid expansion of the central vacuole. In the *yuc8* mutant gametophytes showing defects corresponding to class 2, we observed an unexpanded embryo sac with a small vacuole in the center (Fig 2D and 2E), indicating that rapid growth of embryo sac is compromised. Another ~15% of gametophytes showed defects corresponding to class 3, in which the two polar nuclei were found at either end of the central vacuole (Fig 2F and 2I). This phenotype is clearly distinct from that of the previously reported “unfused polar nuclei” phenotype, where the 2 polar nuclei remain in close proximity at the micropylar end just outside the central vacuole (*e*.*g*. Pagnussat et al. 2005 [20]). Embryo sacs with aberrant polar nuclear migration often had a very well developed vacuole as well as the correct number of nuclei, suggesting that the nuclear migration defect is not a consequence of the defects in vacuole formation or mitotic divisions.

**Fig 4:**
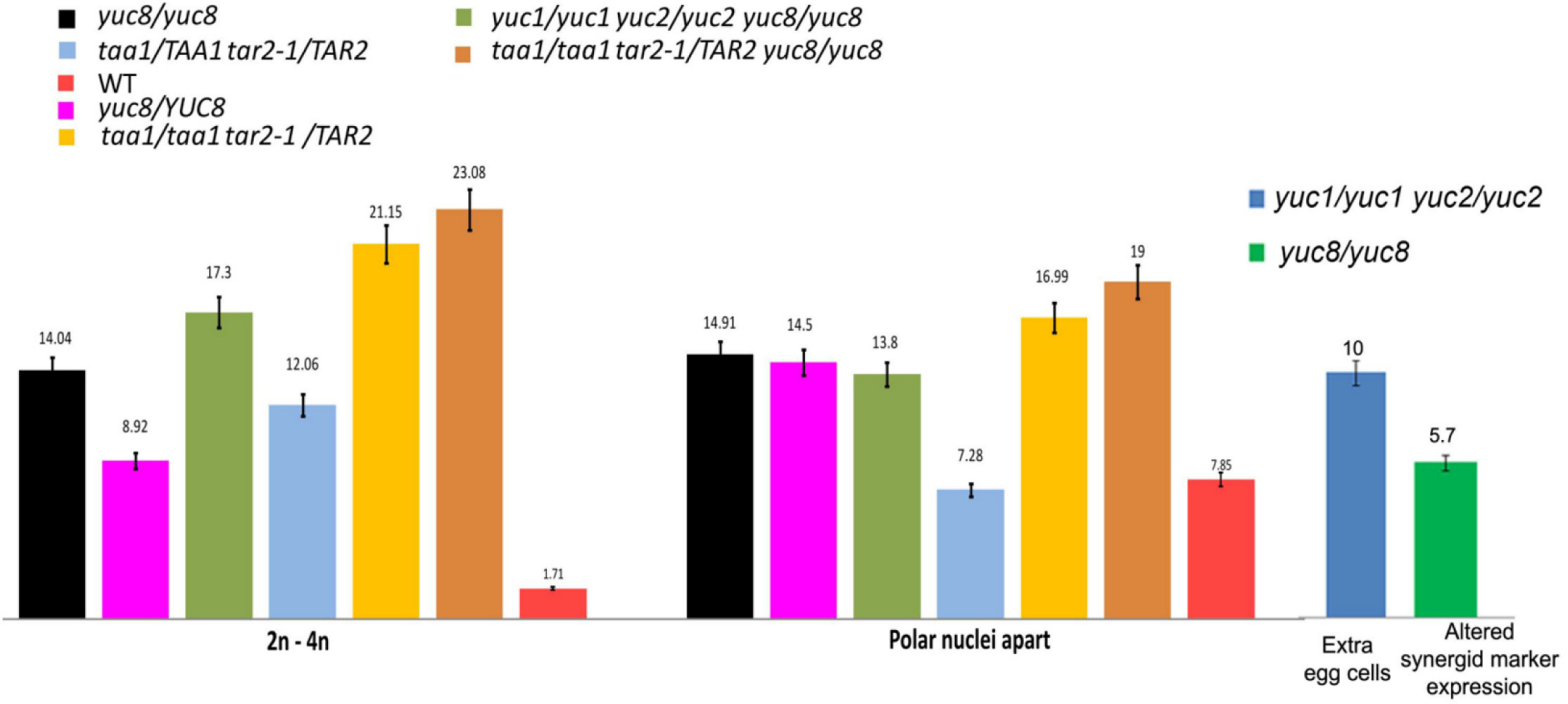
Bar diagram indicating percentage of aberrant embryo sacs in various mutant backgrounds. See **S1 Table** for details. 2n-4n indicates the percentage of gametophytes arrested with 2, 3 or 4 nuclei in an embryo sac that corresponds to a FG6 ovule. “Polar nuclei apart” refers to the percentage of gametophytes with polar nuclei arrested at either end of the central vacuole in a FG7 embryo sac. Extra egg cells and abnormal expression of syn marker were studied in FG7 embryo sacs.

To ascertain whether these functions of *YUC8* are gametophytic or sporophytic, we also undertook observations on *yuc8/YUC8* heterozygous plants. As expected from a gametophytic function for *YUC8*, we observed defects similar to that of the *yuc8* homozygous mutant in the *yuc8*/*YUC8* plants as well, although at a lesser proportion. Compared to *yuc8* homozygous mutant, the *yuc8*/*YUC8* plants showed 8.92% (*vs*. 14% in *yuc8/yuc8*, p-value = 0.08734) ovules with 2 to 4-nucleate embryo sacs at FG5 stage, roughly half of what is observed in the homozygous mutant (Fig 4, S1 Table). Also, 14.50% of gametophytes showed phenotypes corresponding to class 3.

Because auxin produced by *YUC1* and *YUC2* might be alleviating the effects of the *yuc8* mutation, we constructed a *yuc1 yuc2 yuc8* triple mutant by double recombination, as *YUC8* lies between the *YUC1* and *YUC2* genes on Chromosome 4 (see Materials and Methods). The *yuc1 yuc2 yuc8* triple mutant phenotypes were similar to those of the *yuc8* mutant alone, and we did not obtain any additive phenotype, suggesting that *yuc8* is epistatic to *yuc1 yuc2* (Fig 4, S1 Table).

To determine if the embryo sac phenotypes observed are terminal, we emasculated *yuc8* mutant plants, and the unpollinated pistils were cleared after 24hrs. We detected a 12% frequency of ovule abortion in the emasculated plants; the aborted ovules displayed a degenerated material in the place of a mature embryo sac. A wild-type pistil at a similar stage shows only a maximum of 3% ovule abortion. However, the 12% frequency of aborted ovules in the *yuc8/yuc8* mutant is significantly less than the percentage of gametophytes showing visibly defective development in the *yuc8/yuc8* mutant (29%; S1 Table, S3 Fig). Therefore, it is likely that a significant fraction of the mutant gametophytes with developmental defects at the earlier stages were able to recover and form mature functional embryo sacs.

Next, we investigated possible alterations in the gametophytic cell specification in the *yuc8* mutant using the FGR6.0 marker line (see materials and methods), which specifically labels egg cell in red, central cell in yellow, and synergid cell in green. In the wild-type gametophytes we never observed any mis-expression of cell type specific markers. Although no misexpression of the markers was detected in the *yuc8* homozygous mutants, the position of one of the synergid cell´s nucleus was occasionally observed at a chalazal position, resembling the morphology of an egg cell (Fig 3E).

### The *TAA* genes for auxin biosynthesis are expressed during female gametophyte development and required for mitosis and nuclear migration

Analysis of promoter activity using *pTAA1*::*GFP* and *pTAA1*::*GUS* lines indicated strong expression of *TAA1* at the micropylar pole of the embryo sac from FG3 onwards (Fig 1C and S2G—S2I Fig). *TAR2* was also found to be expressed at the micropylar end of the embryo sac, starting a little later than *TAA1*, at about the FG4 stage (S2J—S2L Fig). *TAR2* is expressed in the tip of the inner integument as well from the FG5 through FG7 stages. To explore the gametophytic function of TAA/TAR auxin biosynthetic genes, we examined *taa1*, *tar1*, and *tar2-1* mutants [14]. These single mutants show sporophytic plants of reduced size, but the gametophytes were indistinguishable from those of wild-type plants (not shown). As *TAA1* and *TAR2* show overlapping expression in the embryo sac from FG4 through FG7 we examined *taa1 tar2-1* double mutants. We used the *wei8-1* mutation of *TAA1* as the *taa1* mutant, and the *tar2-1* mutation of *TAR2*, both of which are presumptive null mutants [14]. The *taa1 tar2-1* double homozygous plants did not produce any embryo sacs, being tiny and completely sterile as previously described [14]. So we examined *taa1*/*taa1tar2-1*/*TAR2* plants, which revealed a ~12% (N = 344) reduction in seed set in mature siliques (S3 Fig). DIC microscopy of mature ovules showed that ~ 38% of them contained abnormal gametophytes. 21% of the ovules in the *taa1/taa1tar2-1/TAR2* mutant show 2 to 4 nucleate embryo sacs (Fig 2H). When pistils were examined at earlier stages, we found that while 74% of the embryo sacs have reached an FG4 stage, the remaining ~26% showed only two nuclei inside the embryo sacs (Fig 2G, N = 208). Also, about 17% of the ovules displayed embryo sacs with defects in polar nuclei migration (Fig 2I). Similar phenotypes were observed in *taa1*/*TAA1tar2-1*/*TAR2* double hereterozygous plants as well (S1 Table), indicating that the defects are unlikely to arise from the loss of a sporophytic function. In addition, there is a reduction in the number of defective gametophytes by almost half in the *taa1*/*TAA1tar2-1*/*TAR2* double heterozygotes (S1 Table, p-value = 7.175e-^06^), as compared to the *taa1/taa1tar2-1*/*TAR2* plants. Taken together, these observations suggest that expression of *TAA1* and *TAR2* inside the gametophyte is an important requirement for its normal development.

The phenotypes observed in the female gametophytes of *taa1/taa1 tar2-1/TAR2* plants are similar to those observed in the *yuc8* mutant, but at a higher frequency. A triple mutant was generated by crossing *yuc8*, *taa1* and *tar2-1* mutants. Because *YUC8* and *TAR2* are linked genes on chromosome 4, *yuc8 tar2-1* recombinants were first recovered in the F2. Subsequently, *yuc8*/*yuc8 taa1/taa1 tar2-1/TAR2* plants were obtained (see Materials and Methods), and examined for embryo sac defects. As expected for proteins that work in the same biosynthetic pathway [18], the triple mutant showed phenotypes comparable to those of *taa1/taa1 TAR2/tar2-1* double mutants, both in severity and frequency (S1 Table) and no significant additive effects due to *yuc8* were observed. Taken together, these results are consistent with a role for auxin synthesis through the *TAA/TAR/YUC* pathway in cell division, cell expansion and synergid specification during female gametogenesis.

### Auxin import carriers are required for embryo sac development

**Fig 5:**
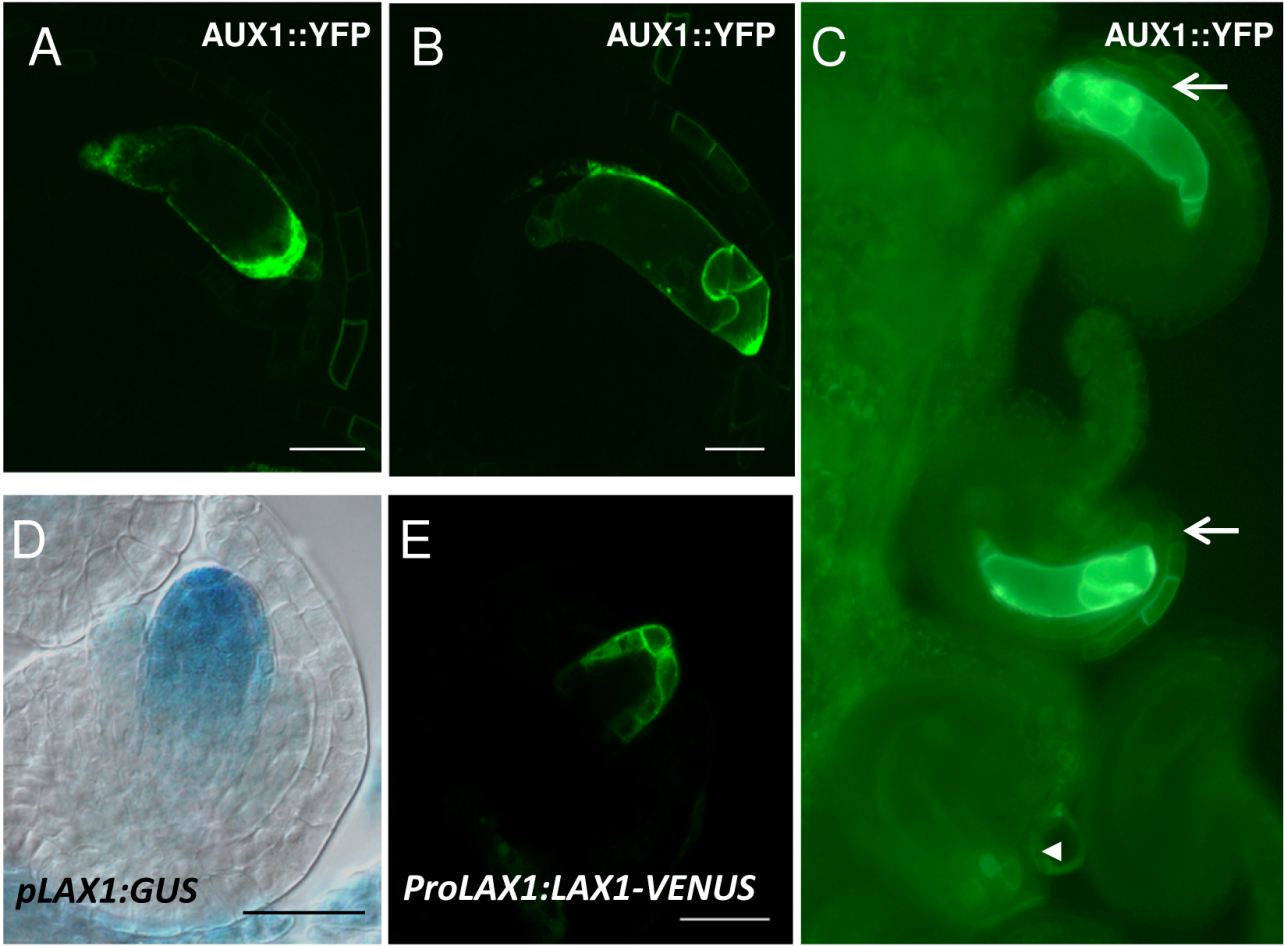
Expression pattern of the auxin influx carriers AUX1 and LAX1 in the developing embryo sac. **A-C**, Detection of AUX1::YFP. **D-E**, Expression of *LAX1* by using the *pLAX1*:*GUS* construct (D) or *ProLAX1*:*LAX1-VENUS* (E). **A**,YFP fluorescence is detected at stage FG4 at the micropylar side of the embryo sac. **B**, After cellularization, at stage FG6, AUX1 is located to egg cell and synergid cell membranes. **C**, Segregation of YFP signal in a line hemizygous for *pAUX1*:*AUX1*:*YFP*. From the four ovules shown, only two present YFP detectable inside the embryo sac (arrows). The arrowhead indicates an ovule in the same pistile without detectable YFP inside the embryo sac. **D,** GUS detection driven by the *LAX1* promoter shows expression at FG1 stage in the functional megaspore and in the nucellus. **E**, Expression of *LAX1-VENUS* in the sporophytic tissues of the nucellus, surrounding the embryo sac micropylar pole at FG2. Scale bar, 20μM.

Our previous study together with another independent study reported that *PIN1* is expressed in the nucellus of developing ovule primordia, and restricted later to the inner integument of the ovule [5,8]. No expression inside the female gametophyte was detected neither for *PIN1* nor for the other members of the *PIN* family [5,8]. PIN1 localization however, indicates that auxin is accumulated in specific nucellar cells, suggesting that auxin could be transported into the developing embryo sac where might regulate gametophytic progression. Supporting this idea, it was reported that downregulation of *PIN1* results in megagametogenesis arrest [8]. To investigate whether auxin import might also be implicated in female gametophyte development, we studied the expression of *AUX1* and *LAX1*, members of the *AUXIN1/LIKE-AUX1 (AUX/LAX*) family of auxin transporters which are the major influx carriers in *Arabidopsis* [21]. As can be observed in Fig 5, using a *pAUX1*::*AUX1*::*YFP* construct we were able to detect AUX1 from stage FG4 onwards at the micropylar pole of the embryo sac (A-C). After cellularization, at stage FG6, AUX1 is located to egg cell and synergid cell membranes. Moreover, *AUX1*:: *YFP* signal is segregating in plants hemizygous for *AUX1*::*YFP*, confirming its gametophytic expression (Fig 5C). While this study was in progress, AUX1 was independently shown to be expressed inside the female gametophyte and the protein to accumulate in the micropylar pole of the embryo sac [7]. We also examined the expression of the related influx carrier *LAX1* (*LIKE-AUX1 1*) by the use of two constructs: *pLAX1*::*GUS* and *ProLAX1*::*LAX1*::*VENUS*. GUS expression was detected early in ovule development in sporophytic tissues of the nucellus as well as in the embryo sac, indicating activity of the *pLAX*1 promoter both outside and inside the embryo sac (Fig 5D). The LAX1::VENUS signal was visible in the sporophytic tissues of the nucellus, surrounding the embryo sac micropylar pole, but in contrast to p*LAX1*::*GUS* expression, the LAX1::VENUS signal was not detectable inside the embryo sac (Fig 5E). However, we noted that the signal from the LAX1::VENUS fusion was weak compared to the AUX1::YFP fusion even in sporophytic tissues, suggesting that low levels of p*LAX1* expression might not be detectable using this reporter fusion. We were not able detect neither *LAX2* nor *LAX3* expression using the available reporter constructs [21].

To assess the possible functions of these auxin influx carriers and of the other members of the *AUXIN1/LIKE-AUX1 (AUX/LAX)* family *LAX2* and *LAX3* in embryo sac development, we examined single, double, triple and quadruple mutants for these genes. The single mutants for *aux1*, *lax1*, *lax2*, *lax3*, the double mutant combinations *aux1 lax1*, *aux1 lax2* and *aux1 lax3* and the triple mutant combinations *aux1 lax2 lax3* and *aux1 lax1 lax3* showed no phenotypes and were fertile. However, the triple mutant *aux1 lax1 lax2* shows ~ 29% of ovules containing embryo sacs arrested at two nuclear stage. The sporophytic tissues of the ovule look normal (Table 1, Fig 2K). Quadruple mutant *aux1 lax1 lax2 aux1 lax1 lax3* shows a phenotype similar to the one observed for *aux1 lax1 lax2* triple mutant, with ~ 28% of aberrant embryo sacs that collapse at FG2 stage (Table 1, Fig 2L). Moreover, when the *aux1 lax1 lax2* triple mutant combination was heterozygous for the *LAX1* wild-type allele, the arrested embryo sac phenotype was still present but at a reduced frequency (10%; Table 1), indicative that the mutations act on the gametophyte and not on the sporophyte. These results suggest that auxin influx carriers *AUX1*, *LAX1* and *LAX2* are redundantly required for normal female gametophyte development. *LAX3* on the other hand is not required for embryo sac development.

**Table 1:** Quantification of aberrant embryo sacs detected in auxin influx mutants.

Genotype	Number of embryo sacs at stage FG6/7 analyzed	
	aberrant/ total	% aberrant
Col	2/232	0.9
*aux1 lax1 lax2*	59/202	29.2
*aux1 lax1 lax3*	7/211	3.3
*aux1 lax2 lax3*	3/174	1.7
*aux1 lax1/+ lax2*	20/198	10.1
*aux1 lax1 lax2 lax3*	57/201	28.4
*yuc8 aux1 lax1 lax2*	59/205	28.8

### *YUCCA1* over-expression with upregulated auxin response restricted to the embryo sac is sufficient to alter chalazal cell fates

In a previous paper, we reported that overexpression of *YUC1* in the embryo sac was able to alter chalazal cell fates, resulting in cells at the antipodal location exhibiting egg cell or synergid cell attributes [5]. However, as auxin response was also observed in the sporophytic tissues surrounding the embryo sac in the lines analyzed, an indirect effect of auxin affecting embryo sac development from the sporophytic tissues could not be ruled out. In a recent study it was postulated that the effects of YUC over-expression might be due to auxin in the sporophytic cells affecting gametophytic cell-specification via a non-cell-autonomous signal [7]. Moreover, the authors reported that the auxin response signals in wild-type ovules were entirely sporophytic, and not gametophytic. However, the gametophytic expression and phenotypes exhibited by the auxin biosynthetic genes and auxin influx carriers characterized in this study implied that auxin signaling is functioning within the female gametophyte. Therefore, the evidence for gametophytic auxin signaling was re-examined in wild-type plants carrying a *DR5- GFPer* auxin response reporter [22] by confocal microscopy, instead of the cytoplasmic GFP reporter used previously. The ER-localized GFPer reporter reduces the possibility of intercellular movement of GFP, and permits more accurate imaging of the localization of the signal. To aid proper staging of ovules during fluorescent confocal microscopy, we crossed wild-type (WT) plants carrying the *DR5*::*GFPer* construct to a marker line that labels all the embryo sac nuclei, as contains the *AKV-NLS*:*Mcherry-AKVT* construct [23]. Additionally, the amphiphilic styryl dye FM4-64, that produces a bright red fluorescence in membranes, was used to delineate the embryo sac at early stages. As can be observed in S4 Fig and as previously reported [5,8], at FG1 stage, right after meiosis, the *DR5*::*GFPer* signal was detected at the distal tip of the nucellus outside the gametophyte (S4A Fig). This signal is retained exclusively in the nucellus up to the end of FG1. After that, a DR5::GFPer signal starts to appear within the developing gametophyte at FG2 stage and gets more noticeable at FG3 stage, at the micropylar pole (S4B and S4C Fig, S3 Movie). At FG4 stage, the DR5::GFPer signal could be clearly observed inside the female gametophyte at a more central position, which is maintained before fading at maturity (S4D–S4F Fig, S4–S6 Movies). The distribution of auxin response at the micropylar pole of the embryo sac observed at the FG2-FG3 stages using confocal microscopy corresponds to the one previously reported using a DR5::GFP reporter and epifluorescence microscopy until the FG3 stage [5]. From FG4 stage to FG5 however, the signal appears to be discretely localized at a more central position than the micropylar localization we reported in the earlier study. It is important to note that the fluorescence signal does not correspond directly to auxin molecules but indirectly measures the auxin response. The observed fluorescence from the GFPer protein is likely to be a reflection of the distribution of ER in the uncellularized embryo sac, and its utilization here is primarily to confirm the presence of auxin signaling inside the female gametophyte. At the later stages, FG6 and FG7, the auxin signal is weak or absent inside the wild-type embryo sac, as previously reported.

**Fig 6:**
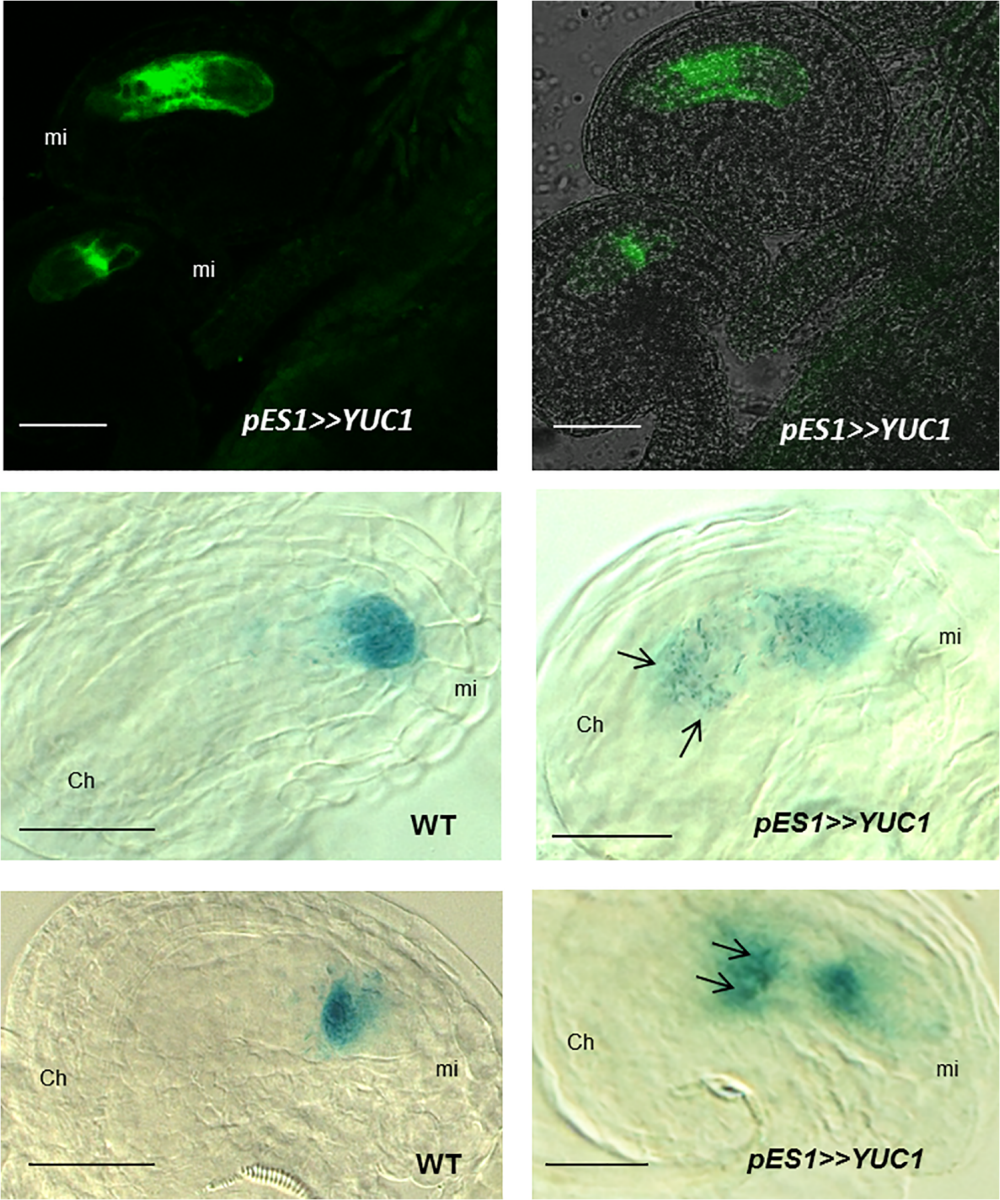
*YUC1* overexpressing embryo sacs show extra egg cells and synergid cells at abnormal positions. **A,** Confocal image showing DR5::GFP activity localized inside the embryo sac of *YUC1* overexpressing female gametophytes. **B**, GFP signal in A is overlapped with a DIC image. **C**, A WT embryo sac showing the expression of a specific synergid marker. **D**, a *YUC1* overexpressing embryo sac showing specification of two extra synergid-like cells at a chalazal position (arrows). **E**, WT embryo sac showing the expression of an egg cell-specific marker. **F**, A *YUC1* overexpressing embryo sac showing expression of the marker in two extra cells at a central position (arrows). Scale bar 20 μM.

To determine whether manipulation of auxin signaling restricted to within the female gametophyte can alter cell fates, a new set of transformant lines were constructed carrying the *Op-LhG4* transactivation system driving the expression of *YUC1* with the *pES1* promoter, which is active from FG1 to FG7 in the whole embryo sac [24]. Transformants were crossed to lines carrying a DR5-GFPer auxin reporter [22], which was preferred over the cytoplasmic GFP reporter used previously to exclude the possibility of intercellular movement. Two independent lines were selected that exhibited auxin response inside the embryo sac without detectable sporophytic signal (Fig 6A and 6B, S5A and S5B Fig). Although the signal was widely distributed along the embryo sac at earlier stages of development (S5 Fig), the signal detected was observed very strong in synergids and egg cell, with less intensity in central cell and in the antipodal cells when embryo sacs reached stage FG6 (Fig 6A and 6B). The fact that *YUC* overexpression triggers a strong auxin response in the embryo sac indicates that not only the rest of the gene products involved in the auxin biosynthesis pathway are present in the female gametophyte, but also that the signaling pathway could be activated in response to auxin. To examine if these embryo sacs that show strong auxin response have cell identity abnormalities, *YUC* overexpressing lines were crossed to egg cell and synergid marker lines. In the F1, 25% of the embryo sacs are predicted to overexpress *YUC1* and 12.5% are predicted to overexpress *YUC1* and to carry the GUS reporter. As can be observed in Fig 6D and Table 2, around 12% of the ovules in the F1 presented abnormal expression of a cytoplasmic synergid marker, which was observed at central and chalazal positions. For WT plants, aberrant patterns were observed in 2 embryo sacs out of 532 ovules analyzed. However, aberrant expression of a different synergid cell marker *pSYN*::*NLS-GUS* was not detected, suggesting that conversion to synergid cell fate might be incomplete in these lines (Table 2). In the case of the egg cell markers, the percentage of aberrant patterns was around 10% for an egg cell nuclear marker and 2.6% for a cytoplasmic egg cell marker (Fig 6F, Table 2 and S5 Fig). In the case of WT plants, no abnormalities were detected in 455 ovules analyzed. All together, these results show that high levels of auxin inside the embryo sac are sufficient for mis-expression of micropylar cell type markers in chalazal cells, and support the model that gametophytic auxin can direct cell specification.

**Table 2:** Expression of cell specific markers in embryo sacs from *pES1*::*LhG4/+; Op*::*YUC1/+; GUS/+* plants.

Pistils studied	GUS positive	GUS negative	GUS positive	Total (100%)	P values *
	WT expression pattern		Abnormal expression pattern (maximum possible = 12.5%^†^)		
*pES1>>YUC1* x cytoplasmic	106 (36.9%)	163 (60.5%)	7 (2.60%)	269 (100%)	0.01399321
Egg cell marker line					
WT x cytoplasmic	117 (45.8%)	138 (54.2%)	0 (0%)	255 (100%)	0.352488
Egg cell marker line					
*pES1>>YUC1* x nuclear	79 (34.80%)	125 (55.06%)	23 (10.13%)	227 (100%)	0.00120237
Egg cell marker line					
WT x nuclear	88 (44%)	112 (56%)	0 (0%)	200 (100%)	0.229299
Egg cell marker line					
*pES1>>YUC1* x cytoplasmic Synergid cell marker line	52 (23.11%)	143 (63.55%)	30 (13.33%)	225 (100%)	< 0.00001
WT x cytoplasmic	244 (45.9%)	286 (53.7%)	2 (0.4%)	532 (100%)	0.196272
Synergid cell marker line					
*pES1>>YUC1* x nuclear Synergid cell marker line	88 (26.99%)	238 (73%)	0 (0%)	326 (100%)	< 0.00001
WT x nuclear	98 (46%)	115 (54%)	0 (0%)	213 (100%)	0.467737
Synergid cell marker line					

* χ2-Test from the expectation that 50% of the gametophytes will be GUS positive, WT expression pattern (i.e. express the cell-specific marker in the correct cells), due to segregation of the GUS reporter.

^†^ Of the total embryo sacs, 25% are predicted to overexpress *YUC1*, and 12.5% are predicted to be both overexpressing *YUC1* and carrying the GUS reporter. Thus, full penetrance would result in 12.5% abnormal marker expression.

## Discussion

### Auxin biosynthetic genes active at specific developmental stages are required for normal female gametophyte development

In this study, we have characterized the expression dynamics and functions of auxin import and biosynthesis in female gametophyte development. S6 Fig summarizes the expression of auxin biosynthetic genes in the female gametophyte, which fall into two sequential groups: The early genes, *YUC1* and *YUC2*, with *YUC2* expression persisting beyond that of *YUC1*; and the mid to late genes, *YUC8*, *TAA1* and *TAR2*, with *TAA1* expression preceding that of *TAR2*. Thus, the syncitial female gametophyte expresses a unique set of genes at different developmental transitions during its short span of development. Moreover, the asymmetrically localized gene expression patterns within the embryo sac suggest that nuclei in the developing embryo sac make different position-dependent decisions at these temporal transitions, implying the existence of spatial cues. This would be possible if the gametophytic nuclei occupy functionally distinct cytoplasmic compartments within the coenocyte, responding to positional signals. Proximo-distal polarity is established early, with the two poles of the embryo sac showing differential expression from the 2-nuclear stage, implying that spatial signals must act by this stage. The first spatial clue perceived by the developing female gametophyte might be sporophytic auxin, which is transported to the nucellus by PIN1 prior to meiosis [5]. In agreement with a critical role of PIN1 early in embryo sac development, it was recently reported that maternal control of *PIN1* is required for female gametophyte development, as PIN1 down-regulation results in embryo sacs arrested at the mono or bi-nuclear stages [8].

Embryo sacs from *yuc1*, *yuc2*, *taa1* and *tar2-1* single mutants were similar to wild-type in all aspects, indicating that these genes act redundantly in the gametophyte. This was confirmed when double mutants for *yuc1* and *yuc2* showed gametophytic phenotypes, although the penetrance was low. In addition, compensation by other auxin biosynthetic genes or by imported auxin might occur when individual genes are mutated. For example, mutants in multiple *YUC* genes do not show significant differences in auxin levels in sporophytic tissues, even though sporophytic phenotypes could be observed [12,25]. Thus, de-repression of one or more of the other *YUC* genes that are normally not expressed in female gametophyte development, or the auxin transporters, might be compensating for the absence of *YUC1* and *YUC2* in *yuc1 yuc2* mutant embryo sacs. *YUC8* expression is the most delayed of the gametophytic *YUC* genes. The *yuc8* mutant displayed distinct growth and developmental defects during embryo sac development, consistent with a requirement for local auxin biosynthesis during megagametogenesis. The percentage of defective embryo sacs in *yuc8/YUC8* was reduced to nearly a half that observed in the homozygous *yuc8* mutant, which is consistent with gametophytic segregation of *YUC8*. The *taa1/taa1tar2-1*/*TAR2* mutant also exhibited gametophytic defects similar to those observed in the *yuc8* mutant, although at higher frequencies. These results strongly suggest that local auxin biosynthesis through the *YUCCA/TAA* pathway is essential for different key processes that take place during embryo sac development. The most frequent defects observed in *yuc8* and *taa1/taa1tar2-1/TAR2* mutant embryo sacs were slower nuclear divisions, reduced cell expansion, and failure of the polar nuclei to migrate towards each other. During the early phase of development corresponding to stages FG3 and FG4, the coenocytic embryo sac undergoes a rapid expansion accompanied by the growth of the central vacuole. Auxin is known to induce rapid cell expansion in sporophytic tissues such as stem, coleoptiles or hypocotyls within minutes of treatment [26]. In addition, we also found that in both the *yuc8* and the *taa1/taa1tar2-1/TAR2* mutant embryo sacs, the size of the vacuole was highly reduced compared to the wild-type counterpart. These observations suggest that the auxin present within the gametophyte at FG3 to FG4 transition (S4 Fig) could be a trigger for the rapid expansion and growth of the embryo sac. The size of the embryo sac is not affected in *yuc1 yuc2* mutants, presumably because these genes are active at earlier stages (FG1 to FG3) prior to the transition to rapid enlargement. The fact that *yuc8* mutants show a phenotype less severe than the *taa1 tar2* double mutant might be accounted for by redundancy with other *YUC* genes that might be expressed at low levels, and not detected by reporter fusions due to technical limitations. Alternately, or additionally, compensatory mechanisms might result in the upregulation of other *YUC* genes in the *yuc8* mutant embryo sacs. In addition to the defects in vacuole formation, the *yuc8* and the *taa1 tar2-1* mutant gametophytes were also asynchronous in post-meiotic mitosis, and 3-nucleate embryo sacs were observed while the WT is at the 4–8 nucleate stages. This is likely due to the effect of auxin on cell cycle progression. For example, the auxin inducible gene *ARGOS* prevents the degradation of CYCLIN D3 when over-expressed, thereby extending the proliferative period [27]. Also, the synergistic action of auxin and cytokinin has been shown to be required for the expression of the cell cycle genes *CDKA*, *CYCD3* and *CDKB1*.*1* in Arabidopsis leaf calli and Tobacco BY2 cells [28].

### Auxin influx genes are required for early growth of the female gametophyte

Auxin influx carriers are also required for normal embryo sac development. The triple mutant *aux1 lax1 lax2* show embryo sacs arrested at FG2 stage, while the development of the ovules is not affected (Table 1, Fig 2). Moreover, the frequency of defective embryo sacs in triple mutants heterozygous for the wild-type *LAX1* allele is approximately half that of triple homozygotes, consistent with gametophytic defects. Double mutant combinations however are fully fertile. These results indicate that auxin influx carriers *AUX1*, *LAX1* and *LAX2* have overlapping functions early in embryo sac development and that their activity is essential for normal female gametophyte development. *AUX1* expression is detectable from stage FG4 and the protein shows a polarized distribution, at the micropylar side of the embryo sac (Fig 2). The expression of other members of the *AUX/LAX* family could not be detected in the developing embryo sac with the fluorescent reporter fusions, although *LAX1* expression could be detected using a GUS reporter. The genetic analysis and triple mutant phenotypes suggest that auxin import is functional at earlier stages, before expression can be detected using these reporter fusions. The expression of *AUX1* is strongly maintained until embryo sac maturity, but remains localized to the membranes of the micropylar cells. Thus, it remains possible that auxin import might be also have additional roles at later stages in embryo sac development during cellularization and cell specification, as was shown for sporophytic development where the AUX-LAX family of auxin influx facilitators is involved in cell type patterning in the apex of the embryonic root in Arabidopsis [29].

The above analysis of the auxin biosynthesis mutants and the auxin influx carrier mutants support a role for gametophytic auxin in promoting development of the embryo sac. Further support is provided by the detection of auxin response signal inside the embryo sac from stage FG2 in this study (S4 Fig). This is contrary to the recent report of an absence of auxin signaling inside the embryo sac [7]. The differences between these studies might arise due to different sensitivities of the reporter constructs used, combined with an overall low level of auxin response signal compared to sporophytic tissues. Using the same degron-based reporter system as in Lutiev et al. [7], which relies on a reduction of GFP fluorescence in the presence of auxin, we were unable to detect even the nucellar auxin signal, which is very clearly observed when using DR5-based reporters ([5,8], and this study). We note that there are also differences between the DR5::GFP signal localization described here with the ER-targeted GFP reporter and our previous report using a cytoplasmic GFP reporter [5], with a more centrally located signal observed at FG4 and early FG5 perhaps reflecting the organization of the ER within the embryo sac at these stages (see Results). The different conclusions reached in previous studies on the presence of auxin within the syncitial embryo sac need to be reconciled with the genetic evidence provided in this study. The simplest and most direct explanation for the mutant phenotypes, segregation analyses and expression data presented here, is that auxin must be acting inside the embryo sac.

### Effect of auxin synthesis genes on cell specification in the female gametophyte

A fraction of the embryo sacs in *yuc1 yuc2* mutants (10%; Figs 3 and 4) showed a shift in cell fate from synergids towards egg cells. However, *YUC1* and *YUC2* show detectable expression only at the early stages (FG1 through FG3), whereas observable cell-specification takes place only at the end of FG5. The loss of these early expressed genes resulting in defects that are manifested at a later stage was therefore unexpected. One possibility is that determinants for embryo sac polarity, *e*.*g*., sequestered factors, are established early, prior to the FG4 stage, and might be induced by the auxin signaling observed at FG2-FG3 stage at the micropylar pole of the female gametophyte and adjacent sporophytic cells. This finding implies that cell-specification might require a more complex mechanism involving other undetermined factors that may be auxin inducible, rather than the simple auxin gradient model that we proposed previously [5]. Another possible explanation for this result might be that there are posttranscriptional mechanisms regulating *YUC1* and *YUC2* mRNA or proteins.

Nevertheless, the previous conclusions that micropylar cell fates are promoted by increased auxin are supported by this study. When auxin distribution is perturbed inside the female gametophyte by overexpressing *YUC1*, embryo sacs show extra cells exhibiting micropylar identities (i.e. synergid and egg cell) at the chalazal pole (Fig 6 and S5 Fig). It has been suggested that the changes in cell-fate by *YUC1* over-expression might be the consequence of auxin that has moved to surrounding sporophytic cells [7]. In this study, transgenic overexpression lines where no auxin signal is evident in the sporophytic cells were utilized (Fig 6 and S5 Fig), yet ectopic expression of egg cell and synergid markers were observed in cells at the location of the antipodals, indicating that chalazal cells had acquired attributes of micropylar cells. These results support the hypothesis that auxin is capable of directing specification of synergids and egg cell fates from inside the female gametophyte.

### Growth dependence on auxin and developmental autonomy of the embryo sac

In this study, the putative functions of auxin at different stages of embryo sac development identified by loss-of-function mutants are: 1. Growth by expansion of the embryo sac. 2. Mitotic divisions during syncitial growth. 3. Specification of future cell types of the individual nuclei after cellularization. These functions were identified by their dependence upon genes for auxin synthesis and auxin import. The functions of auxin in gametophytic development in lower plants have been relatively unexplored, but recent reports have shown that polarization and auxin-mediated patterning mechanisms are present in moss gametophytes. In *Physcomitrella*, *AUX/IAA* and *AFB* mutants defective in auxin signaling exhibit developmental defects at the chloronema-to-caulonema transition [11]. Additionally, it was recently reported that PIN-dependent intercellular auxin transport in *Physcomitrella* mediates growth of filaments and differentiation in leaf-like structures [30,31]. A highly reduced female gametophyte is one of the defining characteristics of flowering plant evolution [32], and represents an evolutionary extreme in terms of the dependence of the gametophyte on the sporophyte. Thus, the findings that the embryo sac relies upon local spatial and temporally regulated biosynthesis and transport of a hormone that is usually transported long distances to its sites of action during sporophtyic development, could reflect the persistence of developmental autonomy in the female gametophyte of flowering plants, a feature that was a part of the free-living ancestral gametophytes.

## Materials and Methods

### Plant materials and growth conditions

All plants were grown in soil (Sunshine Professional Peat-Lite mix 4, Sun Gro Horticulture, Vancouver, BC) in a growth room lit by fluorescent lamps (model TL80; Phillips, Sunnyvale, CA) at 22° ± 3° with a 16 h:8 h light:dark photoperiod. The *yuc* mutants used in this study are all null mutants that were previously characterized and are as described [12,13]. Mutants for the genes *YUC1* (At4g32540) and *YUC2* (At4g13260) were SALK_106293 and SALK_030199 respectively. The mutant for *YUC8* (At4g28720) is a dSpm insertion from the SLAT collection (Sainsbury Laboratory, UK). T-DNA insertion lines CS16413 (*tar2-1/TAR2 wei8-1/wei8-1*), CS16407 (*wei8-1*) and *pTAA1*::*GFP* (CS16432), *pTAR2*::*GUS* (CS16434) are lines that were previously characterized as represent loss-of-function alleles [14] and were obtained from ABRC, Ohio. All genotypes were confirmed by genomic PCR using the following primers: Gene *YUCCA1* WT allele: 5’CCTGAAGCCAAGTAGGCACGTT’3 and 5’CGTTCATGTGTTGCCAAGGGAGATAC’3; T-DNA allele 5’CCTGAAGCCAAGTAGGCACGTT’3 and 5’GGCAATCAGCTGTTGCCCGTCTCACTGGTG’3; Gene YUCCA2 WT allele: 5’CGTCCAATACCTTGAGTCTTACGC’3 and 5’CTGCATACAATCCGCTTTCGC’3; T-DNA allele: 5’ GGCAATCAGCTGTTGCCCGTCTCACTGGTG’3 and 5’ CTGCATACAATCCGCTTTCGC’3. Gene YUCCA7 WT allele: 5’CATGGAGTGGGCTTATCTCTTTG’3 and 5’ACGAAAAACAGAGCACCCTGA3’; T-DNA allele: 5’CATGGAGTGGGCTTATCTCTTTG’3 and 5’GGCAATCAGCTGTTGCCCGTCTCACTGGTG’3. Gene YUCCA8 WT allele: 5’ CTAGTGCTCAACCGTCACAAACCCC’3 and 5’ AACGTTGATTTACCCATTACTTCCCTCGG’3; T-DNA allele: 5’ TACGAATAAGAGCGTCCATTTTAGAGGA’3 and 5’ GAACTGACGCTTCGTCGGGTAC’3. taa1 and tar2-1 mutants were genotyped as described [14]. Promoters used for the p*YUC*::GUS and p*YUC*::GFP fusions were already described [12] and were a gift from Youfa Cheng and Yunde Zhao, pSAV3::GUS, and DR5::GFPer are gifts from Joanne Chory [13], and Klaus Palme [33] respectively. The FGR 1.0 and FGR 6.0 markers were obtained from Rita Groß-Hardt (Groß-Hardt and Völz, unpublished). The nuclear synergid and egg cell markers (pSYN::NLS-GUS and pEC1::NLS-GUS) were obtained from Rita Groß-Hardt (unpublished).

### Cleared whole-mount preparations

Flowers from different developmental stages with at least 40 ovules per pistil were dissected and cleared for 2hrs in Hoyers solution. For GUS staining, developing carpels and siliques were dissected and incubated in GUS staining buffer (5 mM EDTA, 0.1% Triton X-100, 5 mM K_4_Fe(CN)_6_, 0.5 mM K_3_Fe (CN)_6_, and 1 mg mL^–1^ X-Gluc [Rose Scientific] in 50 mM sodium phosphate buffer, pH 7.0) for 24 – 48hrs at 37°C. The dissected pistils were observed on a Zeiss Axioplan imaging 2 microscope under DIC optics. Images were captured on an Axiocam HRC CCD camera (Zeiss) using the Axiovision program (version 4.2).

### Fluorescence microscopy and image analysis

Pistils were dissected in 10mM phosphate buffer on a microscopic slide and immediately observed under fluorescence microscope. Fluorescence detection was done on a Zeiss Axioplan 2 Imaging microscope equipped with epifluorescence illumination and distinct filters for DIC and FITC and RFP using a 63X oil immersion objective. The images were captured with an Axioplan CCD camera using Axiovision software (Zeiss, AxiovisionRel 4.2). Confocal sections were obtained using Olympus FV 1000 Laser Scanning Confocal system. Image processing was done using Imaris image analysis software. All the images were processed using Adobe Photoshop CS3.

### FGR 1.0 and FGR 6.0 marker lines

FGR 1.0 and FGR 6.0 markers were kindly provided by Rita Groß-Hardt. FGR 1.0 comprises of egg cell specific promoter pEC1 [34] fused to NLS::3xdsRED:tNOS, and a central cell specific promoter pDD22 (Steffen et al., 2007) fused to YFP::tNOS. FGR 6.0 is amodified version of FGR1.0 with synergid-specific marker pDD2::NLS::3xGFP::tWUS.

### Construction of mutant combinations

To generate *yuc1 yuc2* double mutants, the *yuc1 yuc2 yuc7* triple mutant [12] was first crossed to *yuc8*, and the F2 segregant sscreened by PCR for *yuc1yuc2YUC7 YUC8* progeny. *YUC2*,*YUC8* and *YUC1* are all on chromosome 4, the genetic distances being approximately 32.5cMfor *YUC2* and *YUC8*, and 7.5cMfor *YUC8* and *YUC1*. Because of the close linkage between*YUC8* and *YUC1*, to generate the triple homozygous mutant *yuc1 yuc2 yuc8*, we first obtained *yuc1yuc8* recombinants. We screened F2 progeny of the triple mutant *yuc1yuc2 yuc7* crossed to *yuc8*,for plants that were *yuc8* homozygous, and which also carried *yuc1* as well as *yuc2*. We further self-pollinated the F2 recombinant (*yuc2 yuc8yuc1/YUC2 yuc8 YUC1; yuc7/YUC7)* to generate the triple homozygous mutant *yuc1 yuc2 yuc8* with WT *YUCCA7* gene. To generate *taa1/taa1 tar2-1/TAR2 yuc8/yuc8* triple mutant, *tar2-1/TAR2 wei8-1/wei8-1* was crossed to *yuc8*. We did not obtain any triple mutant combinations in the F2 due to the genes *TAR2* and *YUC8* being linked on chromosome 4. So, the *taa1/taa1 tar2-1/TAR2 yuc8/yuc8* was obtained by screening the F3 progeny of *taa1/taa1 tar2-1/TAR2 YUC8/yuc8* plants for recombinants between *TAR2* and *YUC8*.

### Constructs and plant transformation

10op::*YUC1* was constructed by inserting *YUC1* cDNA behind an *OP* array (10OP-TATA-BJ36) and subsequently subcloned into the binary vector pCAMBIA 1300 (*CAMBIA*, Canberra, Australia). The plasmid was introduced into *Agrobacterium tumefaciens* strain GV3101 by electroporation into *pES1*:*LhG4* carrying plants with the floral dip method.

## Supporting Information

S1 Fig Diagrammatic representation of the female gametophyte development in Arabidopsis thaliana. Ant, antipodal; Ccn, central cell nucleus; Ec, egg cell; Pn, polar nucleus; Syn, synergid cell.
(TIF)

S2 Fig Expression pattern of various auxin biosynthetic genes in the developing embryo sac., A *pYUC1*::*GUS*; B-C, *pYUC2*::*GUS* D-F, *pYUC8*::*GUS*; G-I, *pTAA1*::*GUS*; J-L, *pTAR2*::*GUS*. A, Embryo sac at FG1 stage showing the expression pattern of *pYUC1*. B, Expression of *pYUC2* in an embryo sac at stage FG2. C, Expression of *pYUC2* in the embryo sac at stage FG4. D, A 2-nucleate embryo sac showing weak expression of *pYUC8*. E, An 8-nucleate embryo sac prior to cellularization, GUS expression can be seen at the micropylar tip of the embryo sac as well as inner integuments. F, A cellularized gametophyte, GUS expression inside the embryo sac has reduced significantly, but the integuments retain a strong GUS signal. G, A 2-nucleate embryo sac showing light expression of GUS, the signal is more concentrated inside the embryo sac. H, An 8-nucleate embryo sac prior to cellularization, a strong signal can be found at the micropylar end of embryo sac. No signal in the integuments as opposed to *pYUC8-GUS* at a similar stage. I, A cellularized embryo sac showing localized GUS signal at the micropylar region, although less intense than FG5 stage. J, A 4-nucleate embryo sac showing very faint GUS expression inside. K, An 8-nucleate embryo sac, prior to cellularization, showing localized GUS activity at the micropylar region and at the tip of the inner integument. L, A mature embryo sac after cellularization showing polarized GUS signal at the micropylar end. Scale bar, 50μM
(TIF)

S3 Fig Sterility in various auxin biosynthetic mutants. Sterility was determined by scoring aborted ovules in a mature silique. taa1/taa1, N = 345. taa1/taa1 tar2-1/TAR2 N = 344. yuc8/yuc8, N = 284
(TIF)

S4 Fig Expression of the synthetic ER-targeted auxin reporter DR5::GFPer during female gametophyte development. The ovules analyzed are from wild-type plants carrying the pAKV-NLS:Mcherry-AKVT construct in order to label all the embryo sac nuclei in addition to the DR5::GFPer reporter (A-F). Additionally, the amphiphilic styryl dye FM4-64 was used to delimit the embryo sac at early stages (A-C). A, At FG1 stage, the signal is strongly detected at the distal part of the nucellus, outside the gametophyte. B, at FG2 stage the signal is now detectable inside the developing embryo sac, at the micropylar pole. C, at FG3 a strong signal is detected at the micropylar pole. See also S3 Movie. D, As the embryo sac continues to develop, at FG4 stage the DR5::GFPer signal is now localized at a central position. See also S4 Movie. E, at late FG5, a DR5 signal is associated with the endothelium, while the signal inside the embryo sac appears to be weaker and localized to a more chalazal position. See also S5 Movie. F, After cellularization but before polar nuclei fusion, the signal inside remains weak. See also S6 Movie. Ant, antipodal cells nuclei; Cc, central cell nucleus; Ec, egg cell nucleus; Fg, indicates the female gametophyte; Fm, functional megaspore; nu, nucellus; oi; Syn, synergid. Scale bar: 20 μm.
(TIF)

S5 Fig YUC1 overexpressing embryo sacs show abnormal expression of specific markers. A, Confocal image showing DR5::GFP activity at FG3 stage. B, GFP signal in A is overlapped with a DIC image C, WT embryo sac showing the expression of a nuclear egg cell-specific marker. D, YUC1 overexpressing embryo sac showing expression of the nuclear egg cell marker in three chalazal nuclei, where antipodal cells are usually specified (arrows).
(TIF)

S6 Fig A diagrammatic sketch of developing ovules summarizing the sequential activation of YUC and TAA/TAR genes in the ovule and embryo sac. (TIF)

S1 Movie Segregation of the GFP signal inside the embryo sac in a line hemizygous for *pYUC8*::*GFPer*. (AVI)

S2 Movie Segregation of the GFP signal inside the embryo sac in a line hemizygous for *pYUC2*::*GFPer*. (WMV)

S3 Movie Expression of the synthetic ER-targeted auxin reporter DR5::GFPer at FG3 stage. (AVI)

S4 Movie Expression of the synthetic ER-targeted auxin reporter DR5::GFPer at FG4 stage. (AVI)

S5 Movie Expression of the synthetic ER-targeted auxin reporter DR5::GFPer at late FG5 stage. (AVI)

S6 Movie Expression of the synthetic ER-targeted auxin reporter DR5::GFPer at FG6 stage. (AVI)

S1 Table Frequencies of embryo sac mutant phenotypes in auxin biosynthetic mutants. (DOCX)
