# Supplementary figures and images for: Auxin Import and Local Auxin Biosynthesis Are Required for Mitotic Divisions, Cell Expansion and Cell Specification during Female Gametophyte Development in Arabidopsis thaliana
Source: PLoS One. 2015 May 13;10(5):e0126164. doi: 10.1371/journal.pone.0126164 (PMC4430233; doi:10.1371/journal.pone.0126164)

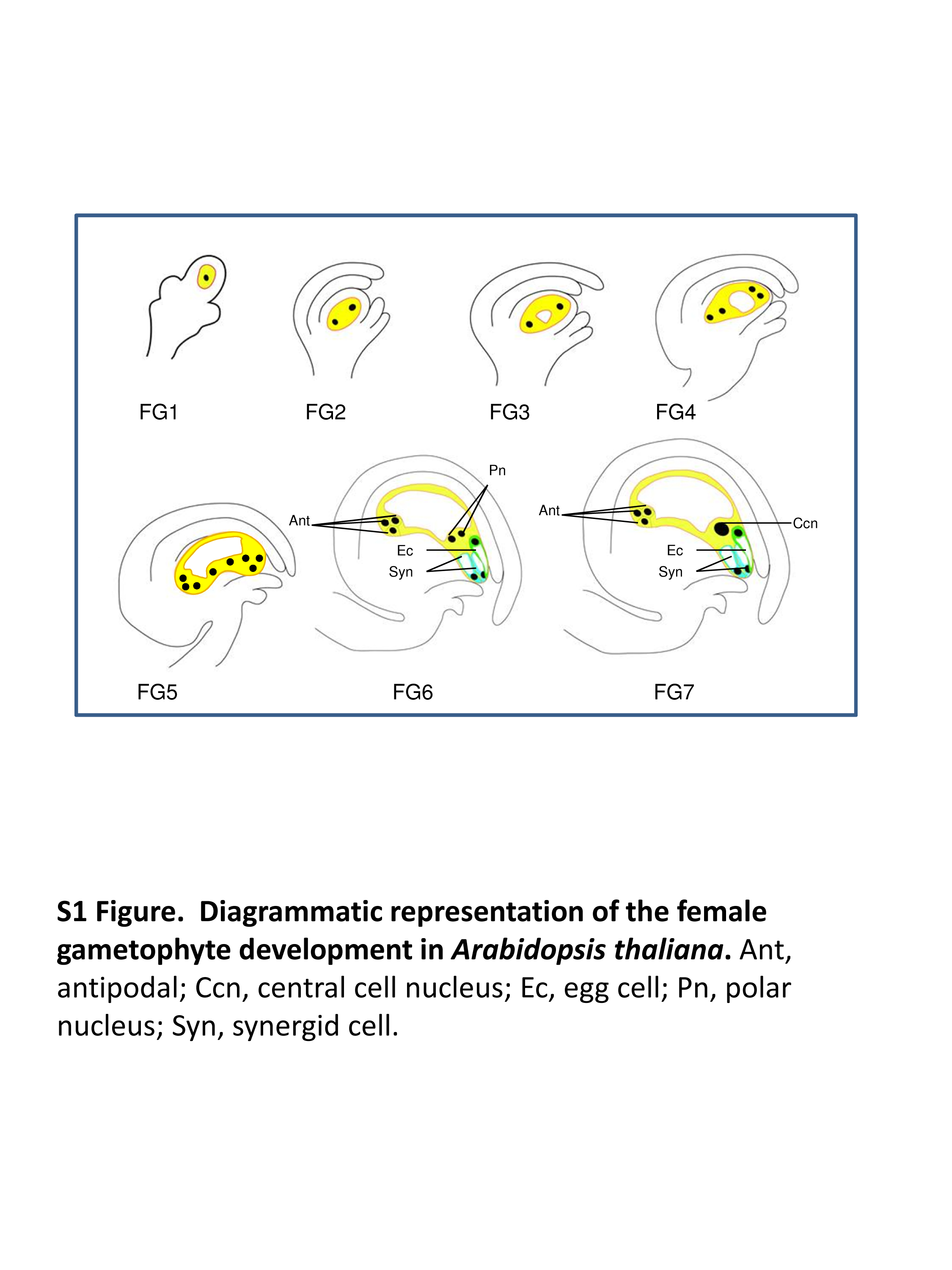

Supplement: S1 Fig — Ant, antipodal; Ccn, central cell nucleus; Ec, egg cell; Pn, polar nucleus; Syn, synergid cell. (TIF) [file pone.0126164.s001.tif]

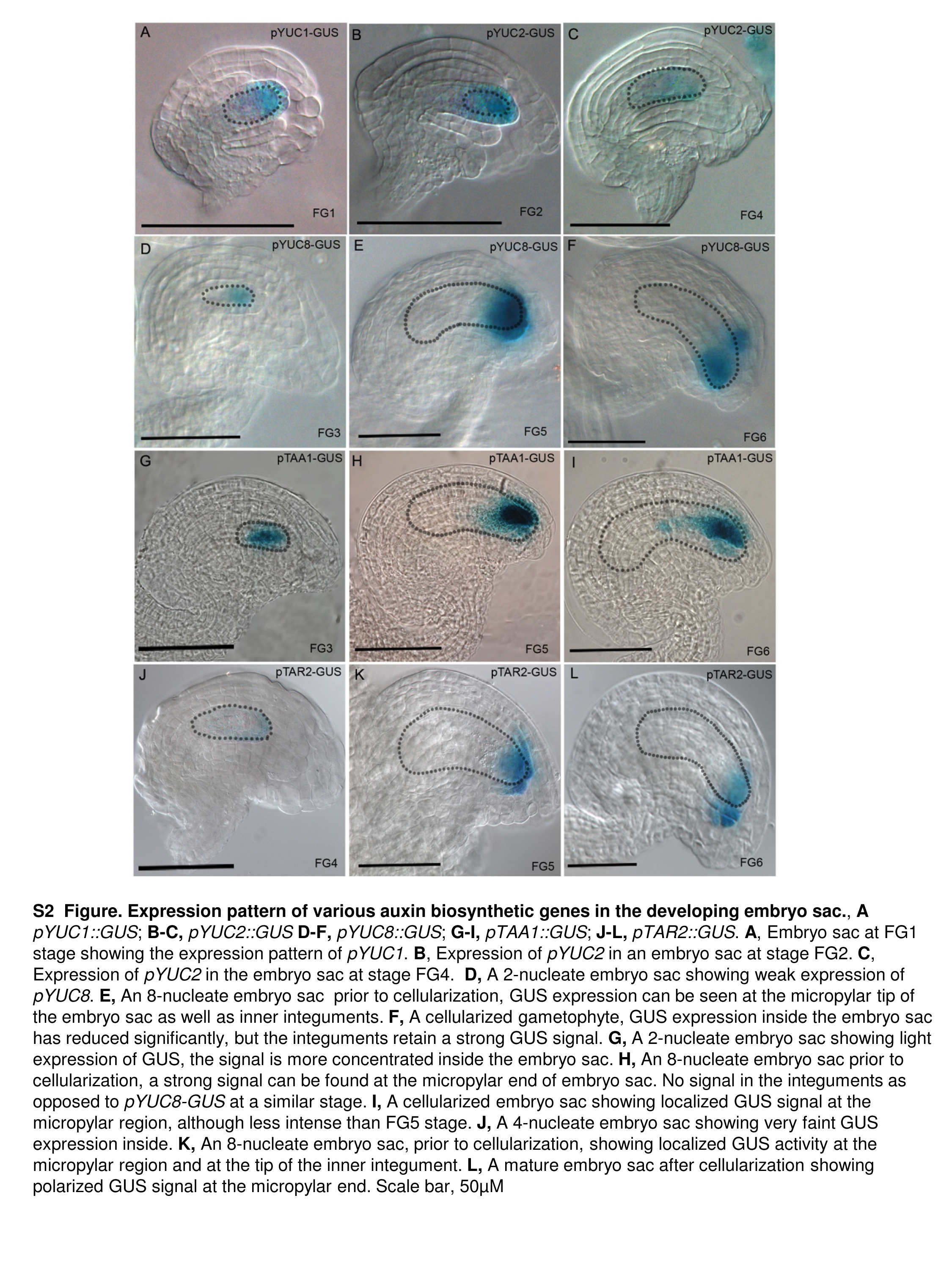

Supplement: S2 Fig — A, Embryo sac at FG1 stage showing the expression pattern of pYUC1. B, Expression of pYUC2 in an embryo sac at stage FG2. C, Expression of pYUC2 in the embryo sac at stage FG4. D, A 2-nucleate embryo sac showing weak expression of pYUC8. E, An 8-nucleate embryo sac prior to cellularization, GUS expression can be seen at the micropylar tip of the embryo sac as well as inner integuments. F, A cellularized gametophyte, GUS expression inside the embryo sac has reduced significantly, but the integuments retain a strong GUS signal. G, A 2-nucleate embryo sac showing light expression of GUS, the signal is more concentrated inside the embryo sac. H, An 8-nucleate embryo sac prior to cellularization, a strong signal can be found at the micropylar end of embryo sac. No signal in the integuments as opposed to pYUC8-GUS at a similar stage. I, A cellularized embryo sac showing localized GUS signal at the micropylar region, although less intense than FG5 stage. J, A 4-nucleate embryo sac showing very faint GUS expression inside. K, An 8-nucleate embryo sac, prior to cellularization, showing localized GUS activity at the micropylar region and at the tip of the inner integument. L, A mature embryo sac after cellularization showing polarized GUS signal at the micropylar end. Scale bar, 50μM (TIF) [file pone.0126164.s002.tif]

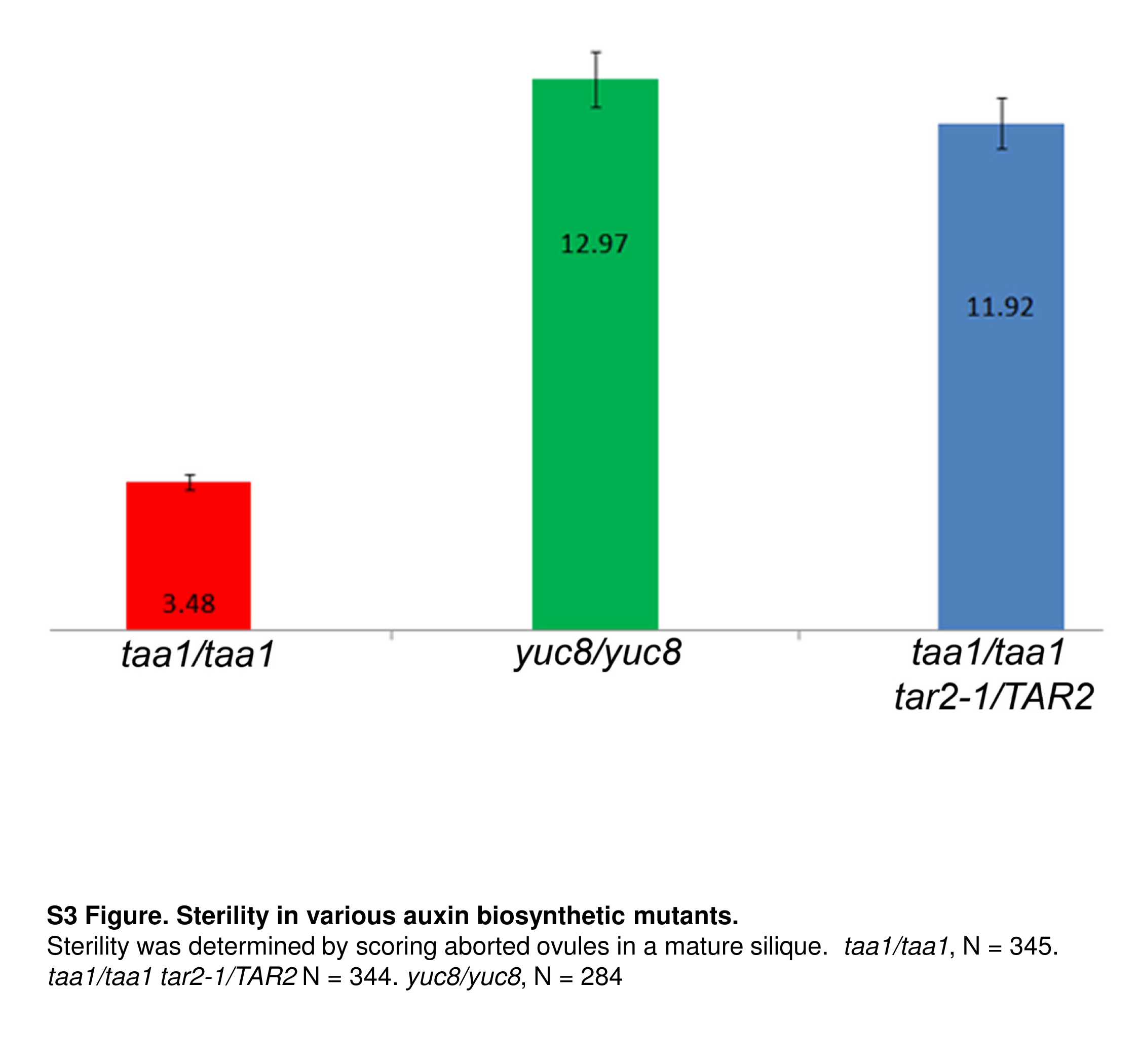

Supplement: S3 Fig — taa1/taa1, N = 345. taa1/taa1 tar2-1/TAR2 N = 344. yuc8/yuc8, N = 284 (TIF) [file pone.0126164.s003.tif]

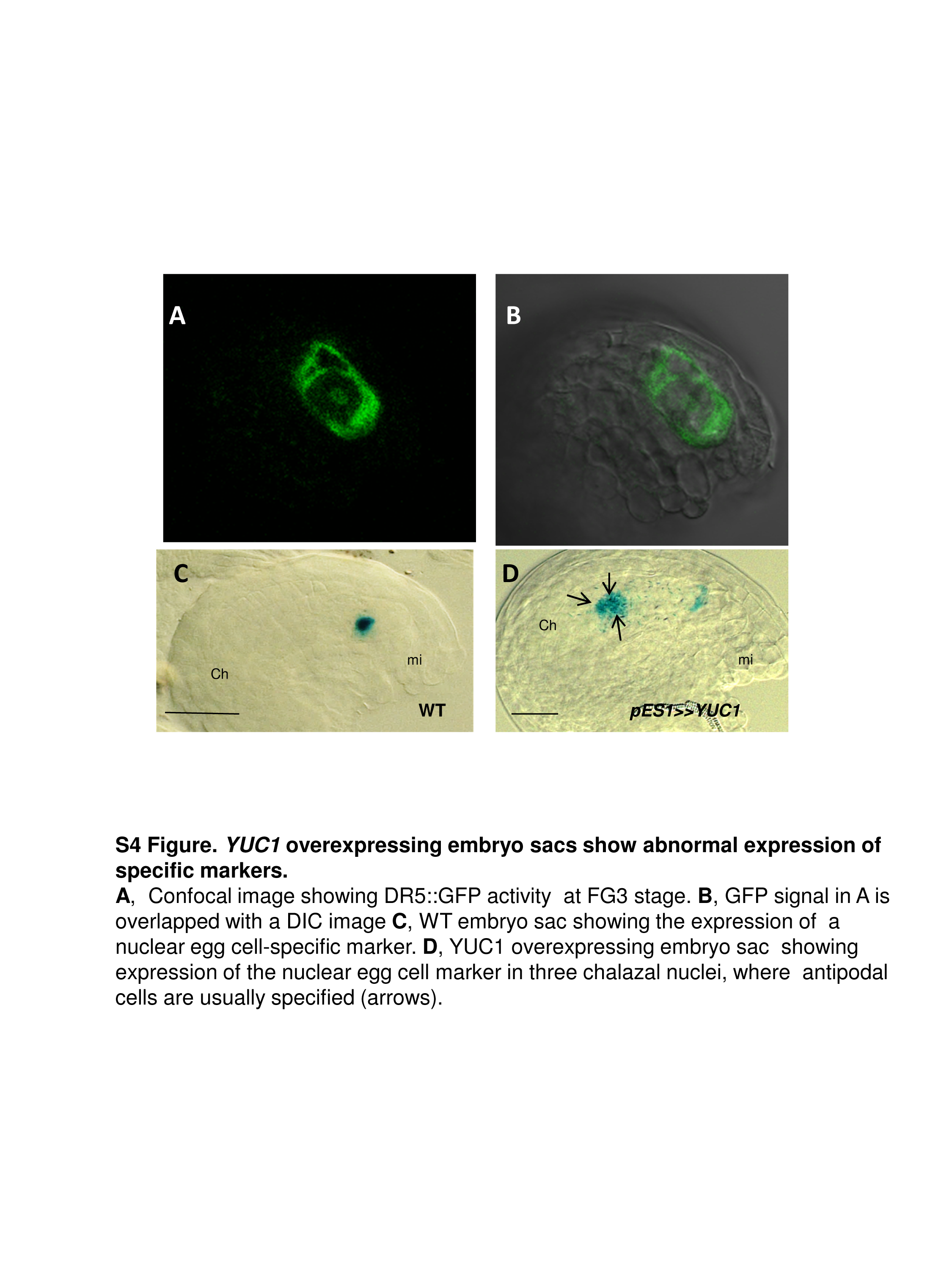

Supplement: S4 Fig — The ovules analyzed are from wild-type plants carrying the pAKV-NLS:Mcherry-AKVT construct in order to label all the embryo sac nuclei in addition to the DR5::GFPer reporter (A-F). Additionally, the amphiphilic styryl dye FM4-64 was used to delimit the embryo sac at early stages (A-C). A, At FG1 stage, the signal is strongly detected at the distal part of the nucellus, outside the gametophyte. B, at FG2 stage the signal is now detectable inside the developing embryo sac, at the micropylar pole. C, at FG3 a strong signal is detected at the micropylar pole. See also S3 Movie. D, As the embryo sac continues to develop, at FG4 stage the DR5::GFPer signal is now localized at a central position. See also S4 Movie. E, at late FG5, a DR5 signal is associated with the endothelium, while the signal inside the embryo sac appears to be weaker and localized to a more chalazal position. See also S5 Movie. F, After cellularization but before polar nuclei fusion, the signal inside remains weak. See also S6 Movie. Ant, antipodal cells nuclei; Cc, central cell nucleus; Ec, egg cell nucleus; Fg, indicates the female gametophyte; Fm, functional megaspore; nu, nucellus; oi; Syn, synergid. Scale bar: 20 μm. (TIF) [file pone.0126164.s004.tif]

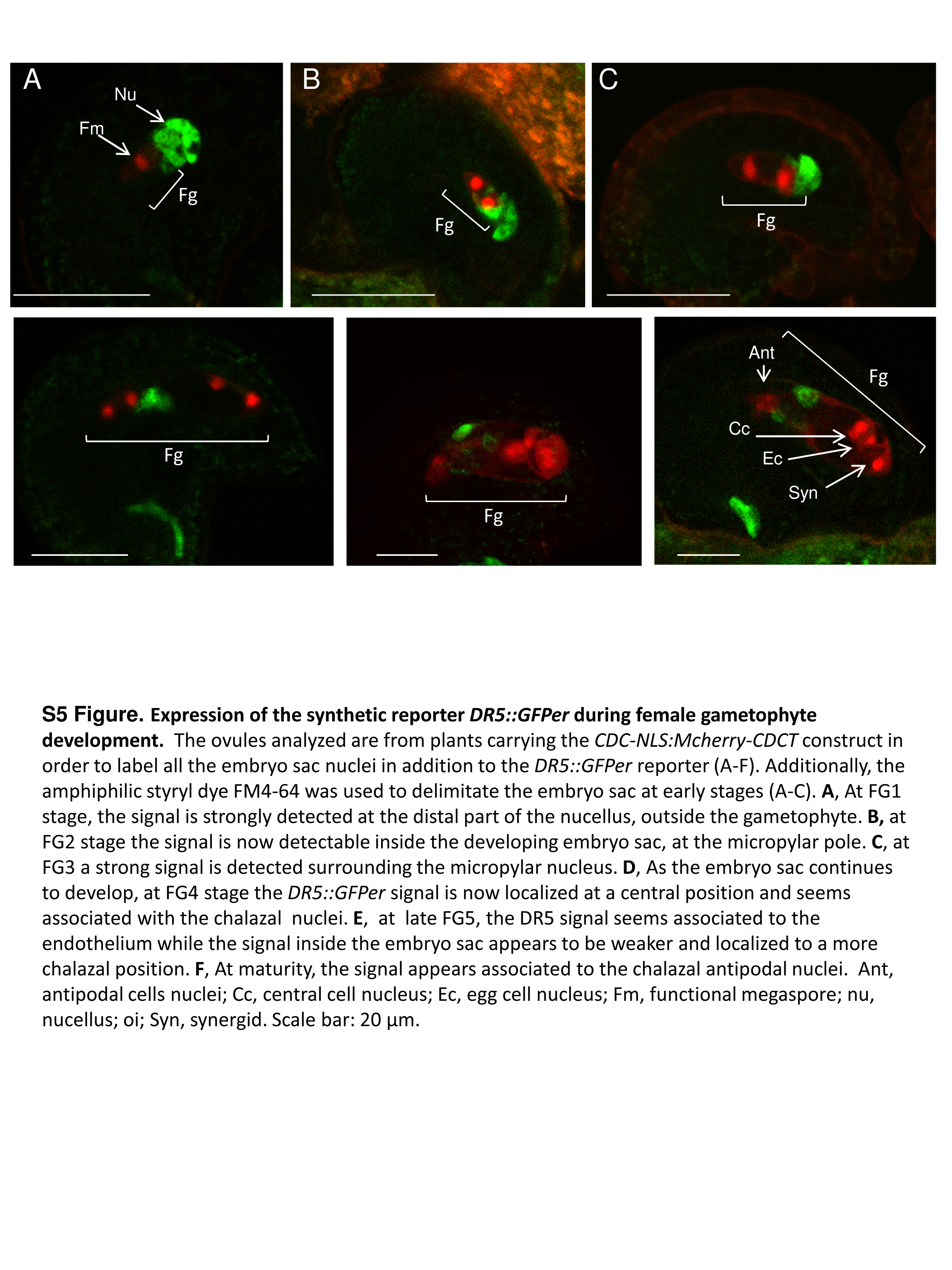

Supplement: S5 Fig — A, Confocal image showing DR5::GFP activity at FG3 stage. B, GFP signal in A is overlapped with a DIC image C, WT embryo sac showing the expression of a nuclear egg cell-specific marker. D, YUC1 overexpressing embryo sac showing expression of the nuclear egg cell marker in three chalazal nuclei, where antipodal cells are usually specified (arrows). (TIF) [file pone.0126164.s005.tif]

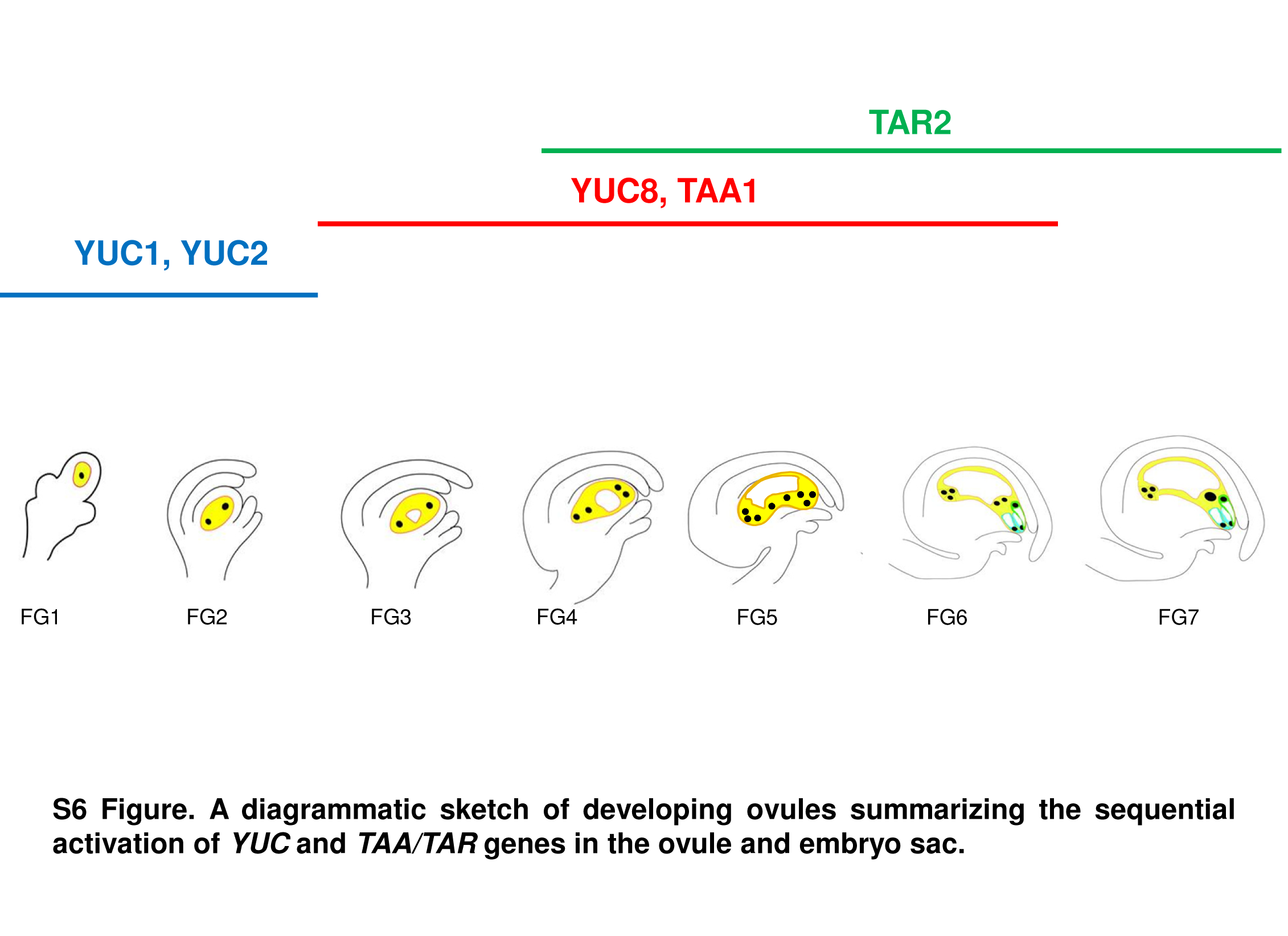

Supplement: S6 Fig — (TIF) [file pone.0126164.s006.tif]
