# Supplementary material for: Auxin Import and Local Auxin Biosynthesis Are Required for Mitotic Divisions, Cell Expansion and Cell Specification during Female Gametophyte Development in Arabidopsis thaliana
Source: PLoS One. 2015 May 13;10(5):e0126164. doi: 10.1371/journal.pone.0126164 (PMC4430233; doi:10.1371/journal.pone.0126164)
Supplement: S1 Table — (DOCX) [file pone.0126164.s013.docx]

| Genotype | 2-4 nuclei | Polar nuclei  apart | Normal | Total* | P-value |
| --- | --- | --- | --- | --- | --- |
| *yuc8/yuc8* | 32  (14.04) | 34  (14.91) | 162  (71.05) | 228 | 1.584e-09 |
| *yuc8/YUC8* | 24  (8.92) | 39  (14.50) | 206  (76.58) | 269 | 7.068e-06 |
| *taa1/TAA1 tar2-1/TAR2* | 23  (12.06) | 15  (7.28) | 168  (81.6) | 206 | 3.367e-05 |
| *taa1/taa1 tar2-1/TAR2* | 66  (21.15) | 53  (16.70) | 193  (61.86) | 312 | 2.2e-16 |
| *yuc8/yuc8 taa1/taa1 tar2-1/TAR2* | 51  (23.08) | 42  (19) | 128  (57.92) | 221 | 2.2e-16 |
| WT | 5  (1.71) | 23  (7.85) | 265  (90.44) | 293 |  |

**S1 Table**. Frequencies of embryo sac mutant phenotypes in auxin biosynthetic mutants

* For *yuc8* homozygous mutant, embryo sacs present at a *3-V* ovule stage were analyzed. For the rest of the mutants, embryo sacs were analyzed in pistils in which at least 50% of the embryo sac reached a mature FG7 stage.

The table shows two major phenotypes from 5 different genotypes examined. The numbers in parenthesis are the percentage of observed phenotypes. The embryo sac phenotypes were scored at the stage when wild-type embryo sacs would be mature *i.e.* corresponding to stage FG7. Ovules were dissected and cleared as described in Materials and Methods for 3-4 hrs. All counting were performed on Zeiss Axioplan 2 microscope under differential interface contrast (DIC) optics after clearing. P-values were calculated using Fischer’s test for *yuc8/YUC8* vs. WT, *yuc8/yuc8* vs. *yuc8/YUC8*, *taa1/TAA1 tar2-1/TAR2* vs. WT, *taa1/taa1 tar2-1/TAR2* vs. *taa1/TAA1 tar2-1/TAR2*, *yuc8/yuc8 taa1/taa1 tar2-1/TAR2* vs. *taa1/taa1 tar2-1/TAR2* respectively. A separate χ^2^ test was used to assess the additive effects of *yuc8* and *taa1 tar2-1* mutations. From the above Table, the penetrance of *taa1 tar2-1* is 77.36% of mutant gametophytes, whereas that of *yuc8* is 46.84%. For the *yuc8/yuc8 taa1/taa1 tar2-1/TAR2* plants, the combined penetrance with no additive effects can be calculated, as gametophytes will be either *yuc8 taa1 tar2-1* or *yuc8 taa1 TAR2,* giving the value of 77.36/2 + 46.84/2 = 62.1%. If there is an additive effect of *yuc8* on *taa1 tar2-1*, the mutant frequency should be larger. As the observed penetrance for embryo sacs arising from this genotype is 42.08% phenotypically mutant gametophytes, there is no additive effect. On the other hand, the reduction in mutant phenotypes compared to the expectation suggests that there might be a suppressive effect on *yuc8* by the *taa1* mutation (χ^2^ = 37.6, P<0.01), possibly due to compensatory up-regulation of *TAR2* or even *TAR1* in the *taa1* mutant background.
